# Linker histone H1 functions as a liquid-like glue to organize chromatin in living human cells

**DOI:** 10.1126/sciadv.aec9801

**Published:** 2026-04-08

**Authors:** Masa A. Shimazoe, Jan Huertas, Charles Phillips, Satoru Ide, Sachiko Tamura, Stephen Farr, S. S. Ashwin, Masaki Sasai, Rosana Collepardo-Guevara, Kazuhiro Maeshima

**Affiliations:** ^1^ Genome Dynamics Laboratory, National Institute of Genetics, ROIS, Mishima 411-8540, Shizuoka, Japan.; ^2^ Graduate Institute for Advanced Studies, SOKENDAI, Mishima 411-8540, Shizuoka, Japan.; ^3^ Department of Chemistry, University of Cambridge, Lensfield Road, Cambridge CB2 1EW, UK.; ^4^ Department of Genetics, University of Cambridge, Cambridge CB2 3EH, UK.; ^5^ Acellera Labs, Barcelona 08005, Spain.; ^6^ Department of Physics, Gandhi Institute of Technology and Management (GITAM) University, Bengaluru 561203, India.; ^7^ Fukui Institute for Fundamental Chemistry, Kyoto University, Kyoto 606-8103, Japan.; ^8^ Department of Complex Systems Science, Nagoya University, Nagoya 464-8603, Japan.

## Abstract

Linker histone H1, the most abundant chromatin protein, condenses chromatin, modulates DNA transactions such as transcription and DNA replication/repair, and participates in differentiation, development, and tumorigenesis. While recent studies indicate that nucleosomes are clustered as condensed chromatin domains in higher eukaryotic cells, how histone H1 mechanically condenses chromatin remains unclear. Here, using a combination of direct visualization of single-H1 molecules in living human cells and multiscale molecular dynamics simulations, we demonstrate that the majority of H1 behaves like a liquid inside chromatin domains, rather than binding stably to nucleosomes as suggested by the traditional model. H1 functions as a liquid-like “glue,” mediating dynamic multivalent electrostatic interactions between nucleosomes within chromatin domains. Consistently, rapid depletion of H1.2 leads to decondensed chromatin domains both in cells and in silico. Our findings suggest that the H1 “glue” condenses chromatin domains while keeping them fluid and accessible, thereby supporting essential DNA transactions.

## Teaser

Single-molecule imaging and MD simulations reveal how histone H1 acts as a liquid-like “glue” to compact chromatin in cells.

## INTRODUCTION

How is the long string of nucleosomes—where DNA is wrapped around core histones (two copies of H3, H4, H2A, and H2B) (*1*, *2*)—organized into chromatin in living cells (*3*, *4*)? A growing body of evidence indicates that chromatin is a highly dynamical and variable structure and is folded irregularly into condensed chromatin domains in higher eukaryotic cells (*5*–*14*). Genome-wide genomics analyses, such as Hi-C (*15*), have also revealed the presence of chromatin domains with distinct epigenetic marks (*16*–*19*).

What factors contribute to the condensation of chromatin into domains? A key contributor is nucleosome-nucleosome interactions mediated by histone tails (*6*, *10*, *20*–*23*), facilitated by cations such as Mg^2+^ (*24*, *25*). Another critical factor is the linker histone H1, which condenses chromatin both in vitro and in vivo (*26*–*30*) and promotes chromatin phase separation in vitro (*23*). H1 is the most abundant chromatin-binding protein (*31*, *32*). It is highly conserved across eukaryotes and forms a large family with 11 distinct subtypes in both humans and mice (*33*).

Structurally, H1 is a tripartite protein consisting of a well-defined and conserved globular domain (GD) with ~80 amino acid residues, flanked by a short, unstructured N-terminal domain (NTD) containing 20 to 35 residues, and a long, intrinsically disordered C-terminal domain (CTD) with around 100 residues (fig. S1A). The CTD comprises about half of the H1 sequence across all subtypes and is highly positively charged, with lysine residues making up ~40% of its amino acid composition. It has a high affinity for nucleosomal and linker DNA and is essential for chromatin condensation (*27*–*30*).

H1 binding to the nucleosome induces structural changes in chromatin, thus regulating transcription and other DNA-dependent processes (*32*, *34*). Consistently, early mouse knockout studies revealed that embryos with reduced H1 levels cease development at midgestation (*35*). H1 functions in gene regulation through three-dimensional (3D) genome organization (*36*), and depletion of H1 leads to perturbations of gene expression, particularly in constitutive heterochromatin in mouse embryonic stem cells (*37*). Furthermore, mutations in genes encoding several H1 isoforms are highly recurrent in B cell lymphomas (*38*). These findings suggest pivotal roles for H1 in development, differentiation, and tumorigenesis through its regulation of higher-order chromatin organization.

How does linker histone H1 condense chromatin into domains within the cell? The classical textbook model has posited that H1 stably binds to the nucleosome dyad (the central base pair of nucleosomal DNA) and stabilizes the 30-nm chromatin fiber (*39*, *40*). Binding modes of H1 around the nucleosome dyad have been well characterized (*41*–*46*). However, recent evidence indicates that the 30-nm fiber is not the fundamental chromatin structure in living cells (*22*, *47*–*49*). Instead, chromatin is organized into irregular, dynamic chromatin domains (*50*). Fluorescence recovery after photobleaching (FRAP) studies have shown that H1 binds to chromatin in a highly dynamic, rather than stable, manner (*51*–*54*). Although most H1 molecules are bound to chromatin at any given moment, this binding is transient, with residence times of ~1 min. These findings underscore the need for a molecular mechanism to explain how H1 interacts with nucleosomes and regulates the chromatin structure.

In addition, chromatin is highly charged, and its structure can vary greatly depending on the surrounding environment, such as the presence of cations, macromolecular crowding (*55*, *56*), or fixation with chemical cross-linking (*57*). There is also a need for technologies to investigate linker histone H1 behavior in chromatin in living cells.

To address these challenges, we used an interdisciplinary approach combining single-molecule imaging and tracking techniques (*58*–*60*), multiscale molecular dynamics (MD) simulations of chromatin (*61*–*63*), and rapid protein depletion technology (*64*). Single-molecule imaging and tracking enable the investigation of molecular behavior in living cells, while multiscale simulations provide mechanistic insights into chromatin organization across different resolutions.

By integrating these approaches, we demonstrate that H1 acts as a liquid-like “glue” for chromatin. In this role, H1 moves dynamically around and between nucleosomes within chromatin, effectively screening electrostatic repulsion between multiple DNA linkers. This dynamic binding enables H1 to condense chromatin while simultaneously fostering DNA fluctuations and a diverse range of internucleosome distances and orientations, thereby promoting an irregular liquid-like chromatin organization.

## RESULTS

### Computational modeling suggests that linker histone H1 acts as a liquid-like “glue” for chromatin

**Fig. 1.**
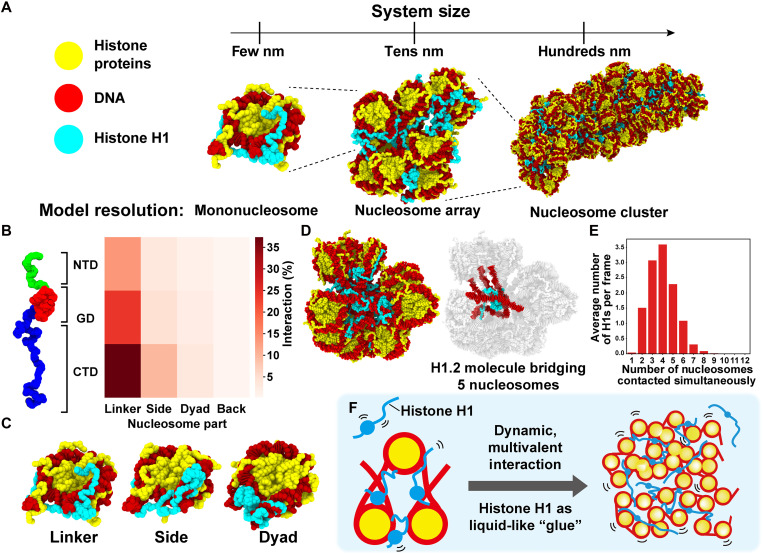
Multiscale computational modeling and a ‘liquid-like “glue”’ model of linker histone H1. (**A**) Scheme for multiscale computational modeling. A single nucleosome, a 12-mer nucleosome array with a nucleosome repeat length (NRL) of 195 bp, and a 108-mer nucleosome cluster (195-bp NRL) are shown. All simulations retain an amino acid–level resolution. (**B**) Region-specific contact map between H1.2 and nucleosomes for a 12-mer nucleosome array (195-bp NRL). H1.2 (*y* axis): NTD, GD, and CTD regions; nucleosome (*x* axis): linker, side, dyad, and back regions. (**C**) Examples of interactions between H1.2 and a nucleosome. (**D**) Representative configuration of a 12-mer nucleosome array (195-bp NRL) with H1.2 (left). One H1.2 can bridge multiple nucleosomes at a time (right) (see also movies S1 and S2). (**E**) Histogram of the number of nucleosomes contacted simultaneously by a single H1.2. (**F**) Model: Linker histone H1 dynamically interacts with nucleosomes through multivalent interactions (left) and has a liquid-like “glue” activity to condense chromatin domains (right).

To gain insights into the behavior of linker histone H1 in chromatin, we first conducted unbiased coarse-grained MD simulations of single nucleosomes and 12-mer nucleosome arrays to examine the binding of H1 in both contexts (movies S1 and S2) (Fig. 1A, left and center). We used our chemically specific coarse-grained model (*65*), in which each amino acid of the histones was represented by one bead and each DNA base pair by one ellipsoid and one point charge per phosphate (see Materials and Methods). We focused on H1.2, one of the most abundant linker histone variants. In these simulations, the H1.2 protein was free to dynamically bind and unbind from the nucleosome (“unbiased MD simulations”), rather than being permanently fixed to the dyad. This dynamical binding/unbinding behavior is regulated by two factors: the interplay of electrostatic and hydrophobic interactions between amino acids and DNA phosphates and the large ensemble of configurations sampled by H1.2.

From these simulations, we calculated the relative frequencies of contacts between the different structural regions of H1.2 (Fig. 1B, left)—its NTD, the GD, and its CTD—and the DNA of the nucleosome. The DNA was categorized into four regions: the side, back, and dyad of the nucleosomal DNA and the linker DNA (Fig. 1C). Although the dyad has long been considered the primary H1 binding site for the GD within the nucleosome, our contact map shows otherwise. In the mononucleosome, all H1.2 domains bind preferentially to the nucleosome side (fig. S1B), while in the 12-mer nucleosome array, all domains more frequently interact with the linker DNA, followed by the nucleosome side (Fig. 1B, right), showing no strong preference for the dyad in either case.

Because unbiased MD simulations can fail to sufficiently explore the conformational landscape of large and heterogeneous systems like chromatin, we next performed temperature replica exchange MD (T-REMD) simulations of 12-mer nucleosome arrays, with one H1.2 per nucleosome, to enhance sampling of H1.2 configurations and assess the impact of H1.2 on internucleosomal interactions (fig. S1C). Analysis of the frequency of interactions between different H1.2 molecules and the various nucleosomes in the array (Fig. 1D) indicated that each H1.2 molecule forms weak and transient contacts with multiple nucleosomes simultaneously (Fig. 1, D and E), continuously exchanging partners (fig. S1, D to F). Most commonly, a single H1.2 molecule contacts the DNA of two distinct nucleosome cores (fig. S1E) and interacts with the linker and nucleosomal DNA across four different nucleosomes (Fig. 1E). In some cases, an H1.2 molecule embedded deep within the nucleosome array bridges as many as eight separate nucleosomes (considering linker and nucleosomal DNA; Fig. 1E). This exceptional capacity of H1 to form multivalent interactions with nucleosomes drives the emergence of a dynamic, irregular, and compact cluster of nucleosomes (fig. S1G), which behaves like a “fluid.” Here, the multivalent, weak, and transient contacts arise from a combination of electrostatic interactions between charged residues and DNA, steric and excluded-volume effects, and the conformational entropy associated with flexible H1 domains.

On the basis of these findings, we propose a liquid-like “glue” model for linker histone H1 function (Fig. 1F). We emphasize that the term liquid-like “glue” does not imply a static or solid adhesive. Rather, it metaphorically describes the dynamic and transient multivalent interactions of H1 with nucleosomes, which enable chromatin compaction while preserving mobility. In this model, each H1 molecule simultaneously engages several nucleosomes through its GD and intrinsically disordered CTD. These interactions are highly transient: The GD and CTD frequently disengage from one nucleosome and rebind to another in an uncoordinated, stochastic, manner (fig. S1F). This lack of coordination is critical: While one domain may transiently unbind, the other typically remains engaged, thereby maintaining the overall association of H1 with the chromatin. Fully coordinated unbinding of all domains is rare—but statistically plausible—and would lead to complete dissociation of H1 from the chromatin. By contacting multiple nucleosomes simultaneously via its different domains and frequently exchanging nucleosome binding partners, H1 facilitates condensation of chromatin while ensuring its fluidity.

### Single-H1 imaging in live human cells

**Fig. 2.**
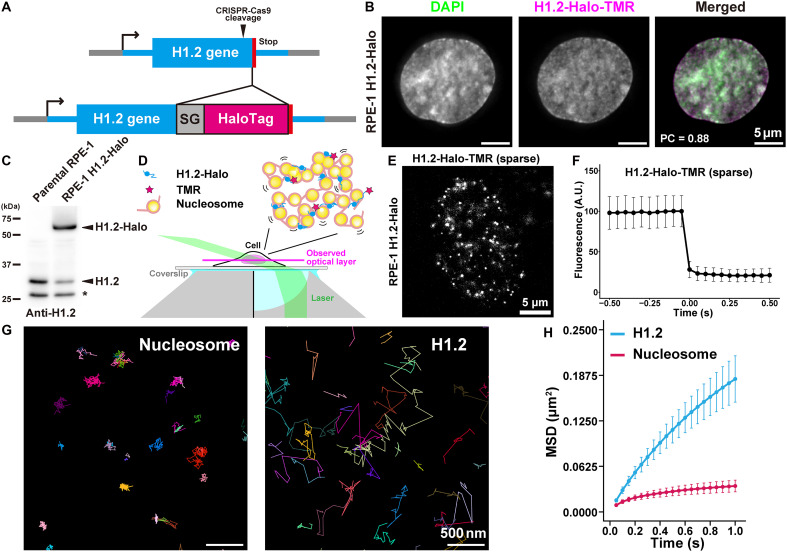
RPE-1 cells stably expressing H1.2-HaloTag and single-molecule H1.2 imaging. (**A**) Schematic of CRISPR-Cas9–mediated genome editing for inserting the HaloTag at the C terminus of the H1.2 gene. SG: linker (GGGGS x3); Stop: stop codon. (**B**) Representative images of RPE-1 cells expressing H1.2-HaloTag (Halo). The localization of H1.2-Halo-TMR is similar to that of DAPI. PC, Pearson’s correlation. (**C**) Western blotting of parental RPE-1 (left) and RPE-1 cells expressing H1.2-Halo (right) using an anti-H1.2 antibody. Note that the untagged H1.2 signal is reduced in RPE-1 cells expressing H1.2-Halo because of monoallelic HaloTag insertion. The asterisk indicates a nonspecific signal. (**D**) Sparse labeling of H1.2-HaloTag-TMR and oblique illumination to illuminate the thin optical layer of the nucleus for single-molecule imaging. (**E**) Representative single-molecule image of H1.2-Halo-TMR in RPE-1. Each white dot represents a single molecule of H1.2-Halo. (**F**) Single-step photobleaching of the H1.2-Halo-TMR signal. Mean intensity plots of 25 dots with standard deviation are shown after aligning the bleaching time point as 0 s. A.U., arbitrary units. (**G**) Representative trajectories of single nucleosomes labeled with H2B-Halo-TMR (left) and H1.2-Halo-TMR (right) for 50 ms per frame. Note that H1.2 moves much more dynamically than the nucleosome. (**H**) MSD plots (±SD among cells) of single-H1.2 (blue) and nucleosomes (H2B-Halo; red) in living RPE-1 cells in a tracking time range from 0.05 to 1 s (*n* = 40 cells for each).

To investigate H1 behavior in chromatin and validate our model, we performed single-H1 molecule imaging in live human cells. We used the CRISPR-Cas9 system (*66*) to introduce a HaloTag sequence at the C terminus of the endogenous histone H1.2 gene in human retinal pigment epithelial (RPE-1) cells (Fig. 2A). The HaloTag allowed for visualization with tetramethylrhodamine (TMR; Fig. 2B) or other Janelia Fluor (JF) dyes (*67*). Cell clones stably expressing H1.2-Halo were labeled with TMR and isolated using fluorescence-activated cell sorting (fig. S2A). Proper monoallelic HaloTag insertion and expression of H1.2-Halo were confirmed by polymerase chain reaction (PCR) and Western blotting (Fig. 2C and fig. S2, B and C). The distribution of H1.2-Halo in RPE-1 cells resembled the pattern observed with 4′,6-diamidino-2-phenylindole (DAPI) staining (Pearson’s correlation = 0.88; Fig. 2B and fig. S2D), indicating genome-wide localization including heterochromatin. Stepwise-salt washing of nuclei isolated from H1.2-Halo–expressing cells demonstrated that the biochemical properties of H1.2-Halo closely matched those of native H1.2 (fig. S2E), confirming appropriate chromatin association. In addition, computational modeling of chromatin incorporating H1.2-Halo produced a region-specific contact map and sedimentation coefficient (*S*) comparable to those obtained without the HaloTag (figs. S1G-5 and S2F), further validating our tagging approach.

Oblique illumination microscopy was used to selectively illuminate a thin layer within a single nucleus (Fig. 2D) (*60*, *68*). This technique, combined with sparse labeling (*60*), allowed us to visualize individual H1.2-Halo molecules as distinct dots (Fig. 2E) and track their movements, recording at 50 ms (movies S3 and S4). As a control, nucleosomes labeled with H2B-Halo in RPE-1 cells were also examined (fig. S3, A to C) (*69*). As in the case of H2B-Halo (*69*), H1.2-Halo dots exhibited single-step photobleaching (Fig. 2F), confirming that each dot represented a single H1.2 molecule labeled with Halo-TMR. The precise positions of individual dots were determined (*70*, *71*) and tracked using TrackMate (*72*) to generate trajectory data. The position determination accuracy values for H1.2-Halo and H2B-Halo were 9.1 and 8.9 nm, respectively (fig. S2G; see Materials and Methods).

Our analysis revealed that the motion trajectories of H1.2 are highly variable, whereas nucleosome trajectories appear more constrained (Fig. 2G). We calculated the displacement (fig. S2H) and mean squared displacement (MSD) (fig. S2I) from the trajectory data. The MSD plots showed that H1.2, on average, exhibits greater mobility than nucleosomes (Fig. 2H). This observation is consistent with previous FRAP studies (*51*, *52*). We also generated a cell line with biallelic HaloTag insertion (fig. S3, D to G) and confirmed that H1.2-Halo in this cell behaves similarly to the one from the monoallele (fig. S3, H to J).

### Machine learning–based H1 trajectory analysis reveals that the majority of H1 behaves like a liquid

**Fig. 3.**
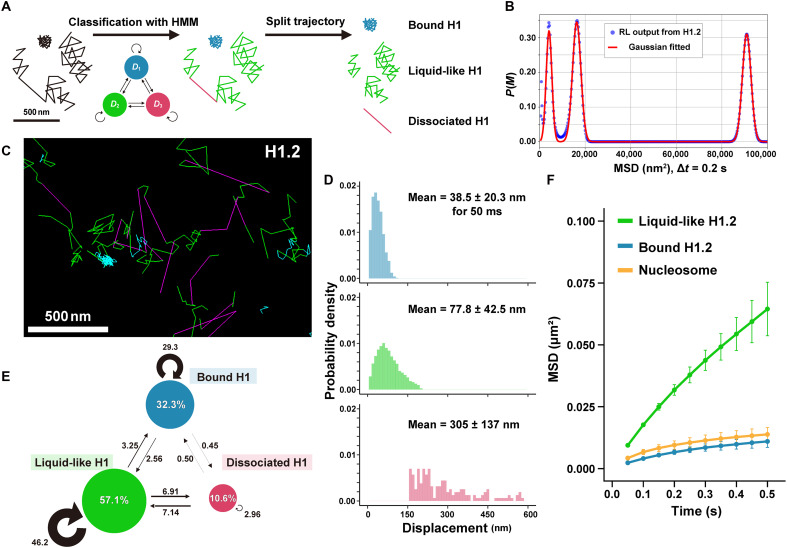
Trajectory analyses of individual H1.2-Halo molecules. (**A**) Scheme of trajectory classification by vbSPT (*73*). On the basis of the hidden Markov model (HMM), trajectories can be classified and split for further analysis. (**B**) Three prominent peaks were obtained from the MSD data of H1.2 by the RL algorithm (*75*). Output from the RL algorithm (blue dots) and the fitted Gaussian mixture (red line). (**C**) Classified trajectories of H1.2. “Bound H1” (cyan), constrained motion from nucleosome binding (Fig. 2G); “liquid-like H1” (green), diffusive motion; “dissociated H1” (magenta), a transient long displacement. (**D**) Displacement distribution histograms (*n* = 40 cells) of classified states for 50 ms. The means ± SD of displacement are at the top of each. (**E**) vbSPT results for H1.2 data (*n* = 40 cells). Diffusion coefficients: *D*_Bound_ = 3.54 × 10^−2^ μm^2^/s; *D*_Liquid-like_ = 1.14 × 10^−1^ μm^2^/s; *D*_Dissociated_ = 1.85 μm^2^/s. Arrows and numbers show the transition direction and percentage between states within 1 frame (50 ms), respectively. Note that the most frequent transition is between the liquid-like and dissociated states. (**F**) MSD plots (±SD among cells) of classified states of H1.2 and nucleosomes (H2B) in living RPE-1 cells over a tracking time range from 0.05 to 0.5 s (*n* = 40 cells for each). Note that trajectories of the “dissociated” state are highly simplified and typically very short in duration (one or two frames at 50 ms per frame), making it difficult to compute reliable MSD values. Therefore, the MSD-versus-time plot for this state is not presented here.

To obtain more detailed information on H1.2 motion trajectories, we used a machine learning–based technique called vbSPT (variational Bayesian single-particle tracking) (*73*, *74*), which can classify H1 trajectories into distinct states using a hidden Markov model (HMM) (Fig. 3A). Each state can be analyzed individually. Through statistical analysis based on the Richardson and Lucy (RL) algorithm, which is a denoising process (deconvolution) (*75*), we identified three distinct states in the H1.2 trajectory data (Fig. 3B) and then categorized the data as blue, green, and red trajectories using vbSPT (Fig. 3C and fig. S4A). The blue trajectories displayed highly constrained motion (displacement: ~40 nm/50 ms; Fig. 3D), similar to nucleosomes (Fig. 2G). The green trajectories showed diffusive motion (~80 nm/50 ms), while the red trajectories represented a transiently “dissociated” H1.2 from chromatin (~300 nm/50 ms) (Fig. 3D). We refer to the fastest population as “dissociated,” meaning transiently dissociated from chromatin that yields near-free diffusion over a 50-ms frame.

After classification, we quantified the percentage of H1 in each state. We categorized 30% of H1, corresponding to the blue trajectories, as “bound” H1 because its motion closely resembled that of nucleosomes (Fig. 3, E and F). A total of 60% of H1, represented by the green trajectories, exhibited an almost linear increase in MSD (exponent α = 0.91) over short timescales up to ~0.5 s (Fig. 3F and fig. S4B), indicative of diffusive motion. This behavior led us to classify this population as “liquid-like” H1, reflecting its dynamic and fluid interaction with chromatin. The remaining 10% was classified as “dissociated” H1. These findings suggest that the majority of H1 behaves in a liquid-like manner. We therefore focused our subsequent analysis on this liquid-like H1 population.

To exclude the possibility that the three states of H1 motion (bound, liquid-like, and dissociated) are specific to H1.2-Halo, we created RPE-1 cells stably expressing H1.0-Halo using a similar strategy to that for H1.2-Halo (fig. S4, C to F) and then investigated the behavior of the H1.0-Halo molecules in live RPE-1 cells (fig. S4G). Consistent with the results for H1.2-Halo, we identified three distinct states in the H1.0 trajectory data (fig. S4, H and I): “bound” (27.7%), “liquid-like” (58.0%), and “dissociated” (14.3%). We concluded that the three states of H1 motion are not variant-specific and that most H1 interacting with chromatin behaves like a liquid.

### H1 diffuses within chromatin domains in a liquid-like manner

**Fig. 4.**
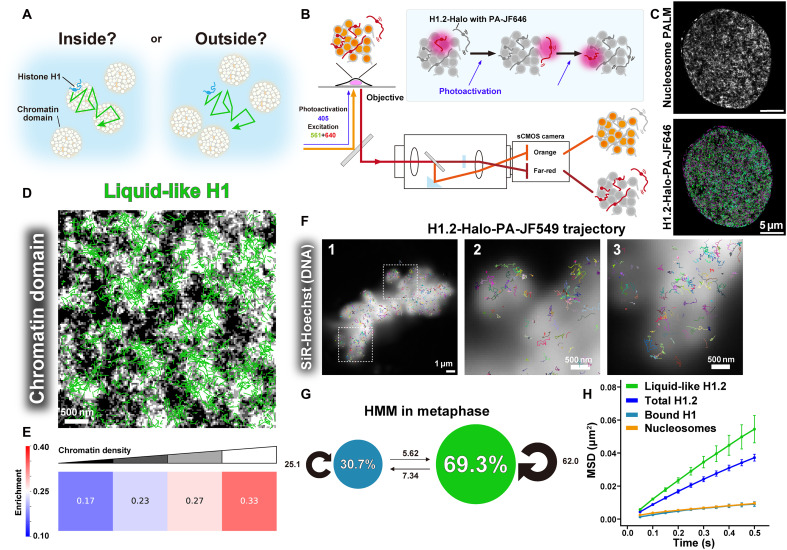
Liquid-like behavior of H1.2 within the chromatin domain and mitotic chromosome. (**A**) Question scheme: Does H1 diffuse like a liquid inside or outside chromatin domains? (**B**) Schematic for dual-color imaging of RPE-1 cells expressing H1.2-Halo and H2B-PAmCherry with a beam splitter system. Box, photoactivation scheme of H1.2-Halo-PA-JF646. The images of H1.2-Halo-PA-JF646 and H2B-PAmCherry were simultaneously acquired with a single sCMOS camera (left half, orange color; right half, far-red color). PA-JF646 and PAmCherry were continuously activated using a very weak 405-nm laser. After PALM reconstruction of H2B-PAmCherry signals, chromatin domains and motion of H1 were visualized at the same time with a nanometer-scale resolution. (**C**) Nucleosome PALM image constructed from H2B-PAmCherry sequential images (50 ms per frame, obtained over 50 s) in an RPE-1 cell (upper) and classified trajectories of H1.2 (bottom) in the same cell. (**D**) Overlap of the PALM image of nucleosomes and trajectories of liquid-like H1. (**E**) PALM images were visualized with four classes of nucleosome density. Enrichment of liquid-like H1 trajectories in dense chromatin regions. A value of 0.25 represents a random distribution of trajectories. (**F**) Overlap of SiR-Hoechst (DNA) signal and trajectories of H1 in metaphase chromosomes (panel 1). Individual trajectories were randomly colored. Panels 2 and 3 are enlarged images from the squared regions in panel 1. H1 shows diffusive movement within mitotic chromosomes. (**G**) vbSPT results for H1.2 data in metaphase (*n* = 10 cells). *D*_Bound_ = 4.63 × 10^−2^ μm^2^/s; *D*_Liquid-like_ = 1.25 × 10^−1^ μm^2^/s. The trajectories consist only of “bound H1” and “liquid-like H1.” (**H**) MSD plots (±SD among cells) of single H1.2 and nucleosomes based on the classified states of H1.2 in living RPE-1 cells in metaphase over a tracking time range from 0.05 to 0.5 s (*n* = 10 cells for each).

We sought to determine whether H1 diffuses like a liquid inside or outside chromatin domains (Fig. 4A). To distinguish between these locations, we visualized chromatin domains in living cells using photoactivated localization microscopy (PALM) superresolution imaging (*6*, *70*) of nucleosomes and combined this with single H1 imaging. We labeled nucleosomes and H1.2 in different colors: H2B-PAmCherry (*6*) for nucleosomes and H1.2-Halo-PA-JF646 (*76*) for H1.2 (Fig. 4B and fig. S5A). Simultaneous observations of nucleosomes and H1 dots were achieved using a beam splitter system (Fig. 4, B and C, and fig. S5B) (*10*, *77*). Chromatin domains were reconstructed from the PALM data, and we collected more than 100,000 H1.2 trajectories from a single cell, which were classified by vbSPT into bound, liquid-like, and dissociated states (movies S5 and S6).

We overlaid the liquid-like H1.2 trajectories onto the chromatin domain image (Fig. 4D and fig. S5, C and D), revealing a clear overlap between the domains and H1 trajectories. Quantitative analysis showed that areas with higher chromatin density (appearing whiter) were more enriched with liquid-like H1.2 (Fig. 4E and fig. S5C). These findings suggest that H1 moves within the chromatin domain in a liquid-like manner, supporting our model that H1 acts as a liquid-like “glue” for chromatin (Fig. 1F).

Our finding that chromatin domains were more enriched with liquid-like H1.2 was further corroborated by observations of H1.2 in mitotic chromosomes (Fig. 4F). These large, condensed chromatin structures with diameters of ~500 to 700 nm (*39*) provided a clearer view of H1 behavior. Dual-color imaging of a single H1.2 molecule and mitotic chromosomes stained by SiR-Hoechst (DNA) clearly visualized that H1 moves within the mitotic chromosome (Fig. 4F and fig. S5E). Using the RL algorithm (*75*), we identified two distinct states in the H1.2 trajectory data from mitotic chromosomes (fig. S5F) and classified them with vbSPT (Fig. 4G). This analysis further affirmed our earlier results by showing that 30% of H1 was in the bound state, 70% exhibited liquid-like behavior (Fig. 4, G and H), and no dissociated H1 was detected.

### Multiscale chromatin modeling supports a liquid-like “glue” property of H1

**Fig. 5.**
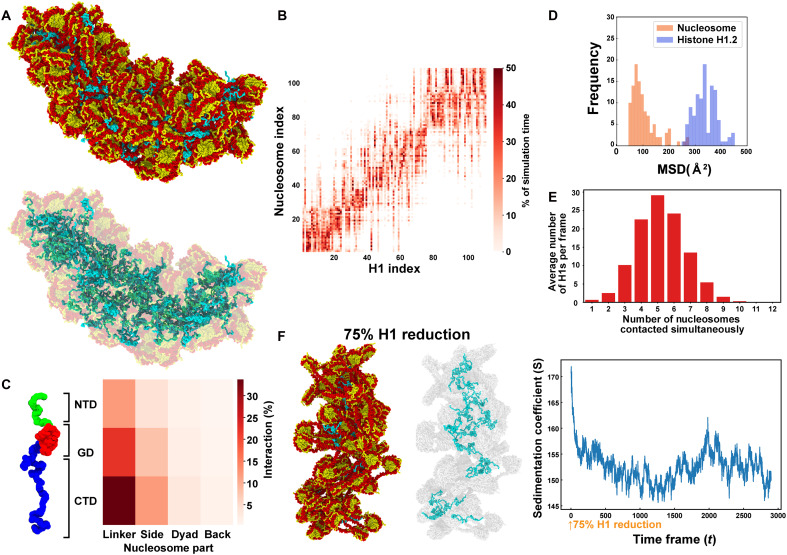
Computational simulation of 108 nucleosomes with H1.2 and glue activity of H1.2. (**A**) Final configuration of an irregular cluster of 108 nucleosomes (195-bp NRL) with 108 H1.2 molecules (also see movie S7). (**B**) Contact map of 108 nucleosomes and 108 H1.2 molecules. Diagonal indicates where H1.2 is located at the beginning of the simulation. Note that H1.2 diffuses among several nucleosomes. (**C**) Region-specific contact map of H1.2 and nucleosome within a 108-nucleosome cluster. (**D**) Histograms for the MSD (Δ*t* = 1 ns) for nucleosomes and H1.2 within a 108-nucleosome cluster. H1.2 has greater mobility than nucleosomes. (**E**) Histogram of the number of nucleosomes contacted simultaneously by H1.2 during the 108-nucleosome cluster simulation in (A) to (C). (**F**) When 75% of H1.2 was removed from the 108-nucleosome cluster, the cluster became decondensed (left, full configuration after reduction; right, only H1.2 is shown). (Right) The sedimentation coefficient (*S*) plot over time is also shown. The sedimentation coefficient markedly decreased after H1 reduction.

To test our model that H1 acts as a liquid-like “glue,” we examined the behavior of H1.2 within a 108-nucleosome domain (one H1.2 molecule per nucleosome) via unbiased MD simulations using our multiscale model (Figs. 1A, right, and 5A; fig. S6A; and movie S7) (*65*). The results demonstrated that H1.2 diffuses throughout the domain, forming transient multivalent interactions by simultaneously engaging multiple nucleosomes (Fig. 5, B to E, and fig. S6B). The region-specific contact map revealed that the nucleosome dyad was the less contacted region for H1.2 (Fig. 5C), consistent with its liquid-like behavior (Fig. 4D). Notably, reducing the number of H1.2 molecules by 75% within the domain (Fig. 5F, left) led to a partial decompaction of the chromatin structure (Fig. 5F, right, and fig. S6C). These findings further support our model that H1 acts as a liquid-like “glue” for chromatin.

### H1.2 is actively involved in chromatin compaction in human cells

**Fig. 6.**
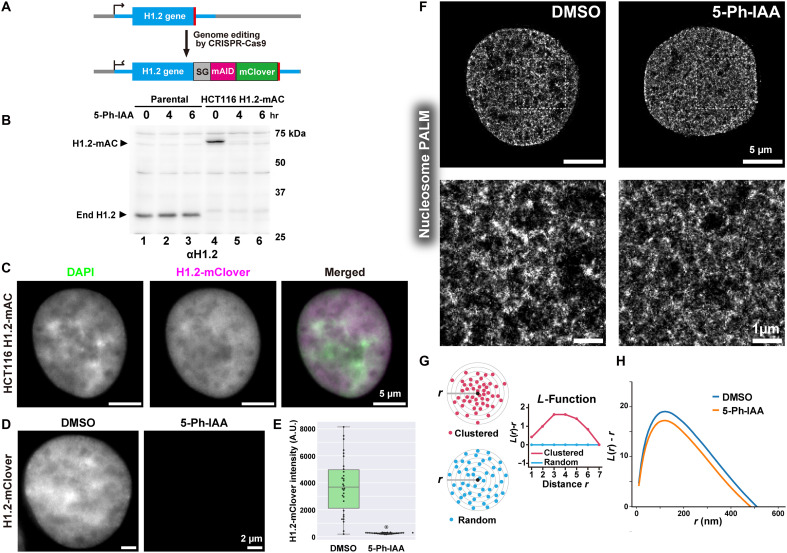
Rapid depletion of H1.2 protein in HCT116 decondensed chromatin domains. (**A**) Schematic of CRISPR-Cas9–mediated genome editing for inserting mAC at the C terminus of the H1.2 gene locus in HCT116 cells expressing OsTIR1(F74G). (**B**) Rapid depletion of H1.2 upon 5-Ph-IAA addition. Western blotting of H1.2 for parental HCT116 and HCT116 cells expressing H1.2-mAC using an anti-H1.2 antibody. Cells were treated with 1 μM 5-Ph-IAA for the indicated time. hr, hours. (**C**) Representative images of FA-fixed HCT116 cells expressing H1.2-mAC. The localization of H1.2-mAC is similar to that of DAPI. (**D**) Representative images of the H1.2-mClover signal in FA-fixed HCT116 cells expressing H1.2-mAC with 0.01% DMSO (left) or 1 μM 5-Ph-IAA for 3 hours (right). (**E**) Quantification of depletion efficiency based on H1.2-mClover intensity in FA-fixed HCT116 cells expressing H1.2-mAC, treated with either DMSO (left) or 5-Ph-IAA (right) (*n* = 30 cells per condition). (**F**) PALM images of nucleosomes labeled with H2B-Halo-PA-JF646 in FA-fixed HCT116 cells expressing H1.2-mAC, treated with either DMSO (left) or 5-Ph-IAA (right). Enlarged views of the squared regions in the upper panels are shown at the bottom. Notably, the H1.2-depleted cell exhibits a more dispersed chromatin domain structure. (**G**) Simplified schematic of *L*-function analysis. Shown are clustered (red spheres, top left) and random (blue spheres, bottom left) particles surrounding the origin point (black sphere). *L*-Function plots for a random pattern (blue) are approximately 0. (**H**) *L*-Function plot of chromatin in DMSO-treated (control, blue) and 5-Ph-IAA–treated (H1-depleted, orange) cells (*n* = 30 cells per condition). *L*-Function values decreased with H1 depletion, indicating chromatin domain decondensation. The statistical significance of this reduction is presented in fig. S7G.

Finally, we investigated whether H1.2, among the H1 variants, plays a direct role in chromatin compaction in human cells. To this end, we used auxin-inducible degron (AID) system 2 (*64*) for rapid H1.2 depletion, combined with PALM superresolution imaging of nucleosomes labeled with H2B-Halo. Using CRISPR-Cas9 genome editing, we inserted a cassette encoding mini-AID (mAID) and the fluorescent protein mClover at the C-terminal site of the endogenous H1.2 gene locus (Fig. 6A) in human colon adenocarcinoma HCT116 cells expressing OsTIR1(F74G) (for details, see Materials and Methods) (*64*) and H2B-Halo (fig. S7A) (*69*). Proper insertion of the tag sequence and expression of mAID-mClover (mAC) were confirmed by PCR (fig. S7, B and C) and Western blotting (Fig. 6B). The distribution of H1.2-mAC in HCT116 cells resembled the pattern observed with DAPI staining, including DAPI-dense heterochromatin (Pearson’s correlation = 0.86; Fig. 6C and fig. S7D), indicating a proper genome-wide localization. Stepwise-salt washing (fig. S7E) also confirmed appropriate chromatin association of H1.2-mAC. In these cells, H1.2 was effectively depleted within 3 hours after adding the auxin analog 5-phenyl-indole-3-acetic acid (5-Ph-IAA) (Fig. 6, B, D, and E, and fig. S7F).

To perform PALM imaging of nucleosomes labeled with H2B-Halo-PA-JF646 (*76*), we fixed cells treated with or without 5-Ph-IAA using formaldehyde (FA) and then reconstructed PALM images of the nucleosomes. The reconstructed images showed chromatin decondensation following rapid H1.2 depletion (Fig. 6F). This finding was further supported by a decrease in the *L*-function plot [*L*(*r*)-*r* versus *r*], which estimates the size and compaction state of nucleosome clusters (or chromatin domains) (Fig. 6, G and H, and fig. S7G) (*6*, *78*). These results indicate that H1.2 is involved in chromatin domain condensation, consistent with our modeling results (Fig. 5F and fig. S6C). Furthermore, they also suggest that tagged H1.2 (with mAID and mClover) retains its functionality.

## DISCUSSION

Using single-molecule imaging, we quantitatively tracked the motions of linker histone H1 and nucleosomes in interphase chromatin and mitotic chromosomes with unprecedented spatiotemporal resolution (10 nm per millisecond scale). By leveraging machine learning–based motion trajectory analysis (*73*, *74*), we uncovered three distinct states of H1 behavior in chromatin. This analysis provides a mechanistic understanding of H1 binding modes in live cells.

**Fig. 7.**
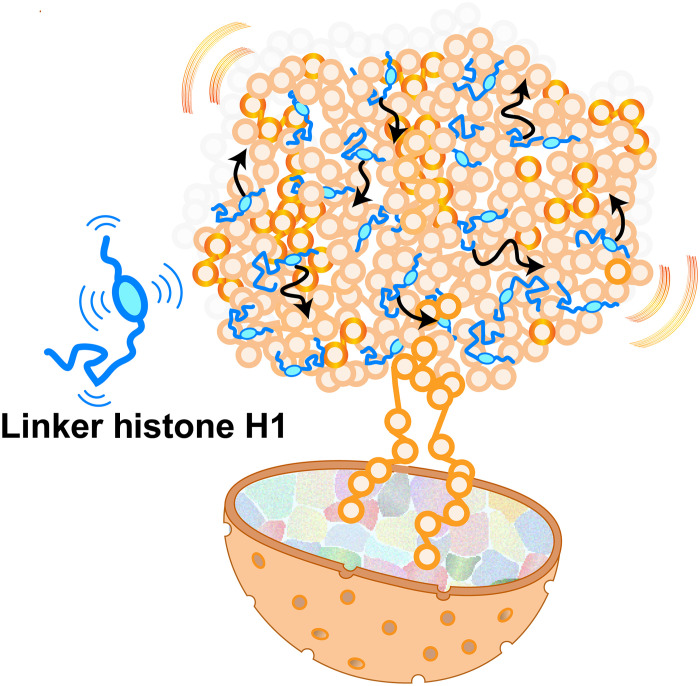
Model figure. Linker histone H1 functions as liquid-like “glue” for chromatin condensation. H1 dynamically interacts with nucleosomes via a positively charged CTD with multivalent interactions. Such liquid-like “glue” activity can condense chromatin while keeping chromatin fluid and accessible. Note: This model is highly simplified—many more H1 molecules exist and move dynamically within chromatin in living cells.

Unexpectedly, 60 to 70% of H1 binds to nucleosomes dynamically and exhibits liquid-like behavior within chromatin domains and mitotic chromosomes. Here, we define “liquid-like” to mean that H1-nucleosomes contacts are maintained but continuously exchanged such that H1 remains associated with chromatin while dynamically moving within chromatin domains or mitotic chromosomes. Given that x-ray crystallography and cryo–electron microscopy studies typically require static and regular structures, this flexible and dynamic state of H1 might not have been well captured. In addition, although cryo–electron tomography of frozen-hydrated cells (*49*) showed “H1-like densities” near the nucleosome dyad, it used subtomogram averaging across many nucleosomes, which might preferentially visualize stably bound H1, while the more flexibly bound population could be blurred out and remain undetected. Our findings indicate that the liquid-like state is the primary binding mode of H1 in cellular chromatin (Fig. 7), consistent with recent evidence that the 30-nm fiber is not the fundamental chromatin structure in living cells (*22*, *47*–*49*). According to the modeling results (Fig. 5E), H1 can interact transiently with several nucleosomes simultaneously, enabling the formation of a liquid network of weak multivalent interactions between H1 and nucleosomes. These findings suggest that H1 functions as a liquid-like “glue” for the chromatin domain, with its positively charged long tails weakly adhering to DNA and acting as a weak glue (Fig. 7).

Our liquid-like H1 model is compatible with the findings of Turner *et al.* (*79*). Turner *et al.* reported that the CTDs of chicken histone H1 remain disordered and dynamic when bound to short double-stranded DNA, forming a liquid-like condensate [H1 condensate was also reported in (*80*)]. This report suggests that the CTDs of linker histones can function as a liquid-like “glue” through dynamic linker histone-nucleosome interactions, which facilitate cross-linking of linker DNA segments (*79*, *81*). Indeed, when a mutant H1, which has a CTD lacking half of its positive charge, was ectopically expressed, the fraction of “liquid-like” H1 markedly decreased (61.5% > 24.8% in fig. S8), suggesting that the CTD’s disorder and high positive charge are crucial for the liquid-like “glue” property of H1.

Our observation also aligns with previous reports using FRAP experiments (*51*–*53*, *82*). Notably, our direct H1 measurements (Fig. 3E) are in good agreement with the predicted fractions of H1 binding modes (slow, 28 ± 6%; fast, 71 ± 5%; free, 0.4 ± 0.1%), which were calculated from FRAP results using a reaction-diffusion model (*53*).

It is also intriguing to discuss the theoretical aspects of the liquid-like “glue” property of H1. We calculated the stable state of H1 using a mean-field model, similar to the polymer model of Flory and Huggins (*83*). This model accounts for the multivalent interactions between H1 and nucleosomes and the entropy gain due to the dynamic binding of mobile H1 molecules. The results predicted that the majority of H1 would adopt a mobile state under nucleosome-dense conditions (fig. S9), indicating that condensed chromatin is more stable with mobile H1 because of its ability to form internucleosomal multivalent interactions. Notably, this theoretical prediction is consistent with our experimental observations and model, which show that ~60 to 70% of H1 behaves like a liquid within chromatin domains and mitotic chromosomes, functioning as a dynamic “glue” (Figs. 3E and 4G).

In contrast, we found that ~30% of H1 stably binds to nucleosomes, which may correspond to “dyad” binding in the classical model. This proportion remained consistent in both interphase chromatin and mitotic chromosomes. Therefore, our study suggests that stable “dyad” binding is not the primary mode of H1 interactions in live cells and occurs mainly as a transient event. “Dyad” binding can condense chromatin by restricting the orientation and flexibility of linker DNA. This raises intriguing questions: Are there specific functions associated with this stable binding mode in cellular chromatin? Could it be genome sequence–specific? Is there a relationship with particular histone variants or modifications? Exploring these possibilities will provide deeper insight into the functional roles of H1 binding in chromatin organization.

Our findings suggest that H1 acts as a liquid-like “glue,” compacting chromatin while maintaining DNA accessibility to proteins, as the nucleosomes within chromatin domains and mitotic chromosomes “glued” together by H1 remain highly mobile. This dynamical mode of compaction may offer several advantages for template-directed biological processes during interphase, such as RNA transcription and DNA replication/repair/recombination. For example, in transcriptional regulation, the liquid-like movement of H1 may facilitate the diffusion of transcription factors and complexes into targets within condensed chromatin domains. Even in mitosis, cells must maintain proper accessibility for large factors such as condensins and topoisomerase IIα, which are involved in chromosome assembly (*84*, *85*). The dynamic, liquid-like behavior of H1 likely enables these proteins to efficiently penetrate into condensed chromatin (*58*, *77*, *86*). The liquid-like H1 binding mode may thus contribute to the assembly of mitotic chromosomes.

Finally, we discuss the limitations of this study. First, H1 behavior in cells might be influenced by multiple additional factors, such as ions [e.g., (*87*)], RNA, and various proteins, including polyanionic chaperones like prothymosin-α, which were not included in our MD simulations. However, the explicit incorporation of these factors is beyond the scope of the present coarse-grained MD framework. The goal of the MD simulations was not to replicate the full biochemical complexity of the nucleus but rather to isolate and test a specific physical mechanism: whether dynamic, multivalent H1-nucleosome interactions are, in principle, sufficient to promote chromatin compaction while preserving configurational heterogeneity and mobility. Second, comparison between experimental trajectories of H1 and MD simulations of H1 could also be informative. However, in the present coarse-grained model, the simulation time (~1 ns) does not quantitatively correspond to experimental timescales (>50 ms), making a direct comparison of diffusion coefficients or state lifetimes nontrivial. A meaningful implementation would therefore require further model calibration and methodological development. This is an intriguing direction for future work.

## MATERIALS AND METHODS

### Multiscale MD simulations of chromatin arrays

The model explicitly represents each amino acid in the histone proteins with one bead centered on its $C_{\alpha }$. Beads corresponding to lysine, arginine, aspartic acid, glutamic acid, and histidine carry the total charge of their atomistic counterparts at pH 7. The relative hydrophobicities and diameters of all amino acid beads were derived from atomistic simulations and experimental data as described in (*65*).

To preserve the secondary structure of the histones within the histone core, we used an elastic network model defined by our atomistic MD simulations of mononucleosomes (*63*), which were based on the 1KX5 crystal structure (*88*). In contrast, the histone tails were modeled as fully flexible polymers, with bonds between consecutive residues maintained via a stiff harmonic potential and no energetic penalty considered for bending or torsion.

DNA was modeled at a resolution of one ellipsoid per base pair and one point charge per phosphate group (*65*). The sequence-dependent mechanical properties of DNA were implemented using a modified version of the rigid base pair model (*65*, *89*), which approximates interbase pair step deformations with a six-dimensional harmonic potential (shift, slide, rise, roll, tilt, and twist) using parameters derived from large-scale atomistic simulations of free DNA strands (*90*).

Electrostatic interactions between all nonbonded beads were approximated using the Debye-Hückel model (*91*), while nonionic associations were modeled using a Lennard-Jones potential (*91*). Parameters for amino acid pair interactions were taken from the Kim-Hummer model (*92*), and amino acid-DNA parameters were derived from atomistic simulations of nucleosomes (*63*). Further details of all model parameters and energy functions are available in (*65*).

For the 12-nucleosome array simulations, we tested two regular nucleosome repeat lengths (NRLs): 165 and 195 base pairs (bp). The initial configurations included one H1.2 molecule bound to each nucleosome in its dyad position. All the simulations were performed for the human H1.2 protein (UniProt Consortium ID: P16403). We defined the N-terminal domain as residues 1 to 38 and the CTD as residues 109 to 213. We used Modeller to create a homology model of the GD of H1.2 with H5 [PDB (Protein Data Bank): 4QLC] as the template. In all the simulations, H1 proteins can bind/unbind dynamically from the dyad position and diffuse around chromatin freely.

For efficient sampling of chromatin configurations, we performed T-REMD simulations at 0.15 M NaCl (Debye length of 8 Å). The number of replicas and spacing were optimized to ensure acceptance probabilities exceeding 30% between neighboring replicas. We required 64 replicas to span temperatures between 300 and 600 K. These simulations were performed in the canonical ensemble (NVT) using the Langevin thermostat (damping time of 10,000 fs) and the velocity Verlet integrator implemented in LAMMPS (*93*). The simulations were run for ~100 million steps using a time step of 40 fs. T-REMD exchanges were attempted every 1000 steps, and simulation snapshots were recorded every 100,000 steps.

For the 108-nucleosome array simulations, we built the system using nine preequilibrated configurations of 12-nucleosome arrays. This is an unbiased simulation run at 0.15 M NaCl (Debye length of 8 Å) and 300 K for illustration purposes.

For analysis, we computed sedimentation coefficients using the HullRad method (*94*), which uses a convex hull model to estimate the hydrodynamic volume of chromatin. Specifically, the hydrodynamic volume is approximated by constructing the smallest convex envelope around chromatin while accounting approximately for hydration effects and solvent interactions. The HullRad method is particularly suited for flexible and irregular structures like chromatin.

### Plasmid construction

Construction of the pX330 CRISPR-Cas9 plasmid (no. 42230, Addgene) expressing guide RNA for H1.2 or H1.0 target sites and the donor plasmids (nos. 247480 to 247483, Addgene) was performed as follows. Gene-specific guide RNA sequences for H1.2 or H1.0 were designed using the CRISPR design website and inserted into the pX330 Bbs I cloning site (*66*). The guide RNA sequences were as follows: for H1.2, 5′-CACCGGGTTGTCAAGCCTAAGAAGG-3′ and 5′-AAACCCTTCTTAGGCTTGACAACCC-3′; for H1.0, 5′-CACCGCTTGCCGGCCCTCTTGGCAC-3′ and 5′-AAACGTGCCAAGAGGGCCGGCAAGC-3′.

For the donor plasmids of H1.2-HaloTag and H1.0-HaloTag, the left and right homologous arms were PCR amplified using KOD FX (KFX-101, Toyobo) from RPE-1 genomic DNA, which was extracted using a Wizard Genomic DNA Purification kit (A1120, Promega). The primer sequences were as follows: for H1.2 of left arm, 5′-AGATCCACCTCCACCTTTCTTCTTGGGCGCTGCCTTCTTAGGCTT-3′ and 5′-GCGGCCGCGGGAATTCCTCCTGCCGCTCCCGCTGC-3′; for H1.2 of right arm, 5′-GAGATTTCCGGTTA-ATAGGCGAACGCCTACTTCTA-3′ and 5′-CTCCCATATGGTCGACGGACAAAATGGCTGGCTAAAAGT-3′; for H1.0 of left arm, 5′-GCGGCCGCGGGAATTCTCCACAGACCACCCCAAGTA-3′ and 5′-AGATCCACCTCCACCCTTCTTCTTGCCGGCTCTCTTGGCAC-3′; for H1.0 of right arm, 5′-GAGATTTCCGGTTAATGACAATGAAGTCTTTTCTT-3′ and 5′-CTCCCATATGGTCGA-CTCGGGAGGTTTTAAGTGGCC-3′.

The HaloTag sequence was PCR amplified from the pFC14A HaloTag CMV Flexi Vector (G965A, Promega) using the following primers: 5′-GGTGGAGGTGGATCTGGTGGAGGTGGATCTGGTGGCGGCGGTTCAGGATCCGAAATCGGTACTGGCTTTC-3′ and 5′-TTAA CCGGAAATCTCCAGAG-3′. The homologous arms and HaloTag fragment were joined using standard overlapping PCR and inserted between the Eco RI and Sal I sites of the pGEM-T (Easy) vector (A1360, Promega) using In-Fusion (639648, Clontech).

To construct the donor plasmid for H1.2-mAC (no. 247486, Addgene), sequences except HaloTag were amplified from the H1.2-HaloTag donor plasmid using the following primers: 5′-TGAACCGCCGCCACCAGATC-3′ and 5′-GCGAACGCCTACTTCTAAAACC-3′. mAC was amplified from the mAC-POLR2A donor (Hygro) (no. 124496, Addgene) with 5′-GGTGGCGGCGGTTCAAAGGAGAAGAGTGCTTGTCC-3′ and 5′-GAAGTAGGCGTTCGCTTACTTGTACCAAGGCCTTCCGTCCATGC-3′. Both fragments were joined using In-Fusion.

For vector construction to transiently express wild-type H1.2-HaloTag (H1.2-WT), fragments were amplified from a donor plasmid of H1.2-Halo using the following primers: 5′-CAGCGGGAGC-GGCAGGAGCAGTCTCGGACATGGTGGCGTTAATTAACCTTAAGTTTACGAGGG-3′ and 5′-CTCTGGAGATTTCCGGTTAATAGACCGGTGCGGCCGCAATCGATCGCC-3′. The amplified H1.2-Halo fragments were joined together using standard overlapping PCR and inserted into the Pac I and Age I sites of pAAVS1-NDi-CRISPRi (no. 73498, Addgene) using the XE cocktail (*95*). The backbone part of the H1.2-WT vector was amplified using 5′-GGATCCGAAATCGGTACTGGCTTTC-3′ and 5′-CTCATCACCAAGGCTGTGGCCG-3′. Then, the synthesized H1.2-Khalf CDS (gBlocks, IDT) and the backbone were ligated using the XE cocktail (nos. 247484 and 247485, Addgene).

### Establishment of stable cell lines and cell culture

To establish RPE-1 cells that stably express H1.2-HaloTag from the endogenous locus, RPE-1 cells at 80% confluence in a six-well dish were cotransfected with 750 ng each of pX330 (with a target gene–specific guide sequence inserted) and the H1.2-Halo donor plasmid using the Neon electroporation transfection system (Thermo Fisher Scientific, MPK1025). Twenty-four hours after transfection, transformants in the six-well dish were transferred to a 10-cm dish and expanded until they reached 5 × 10^6^ cells/ml. To select transfected cells from untransfected cells, a cell sorter (SONY, SH800S) was used. Before sorting, cells were incubated in a medium containing 50 nM TMR-HaloTag ligand (8251, Promega) for 16 hours. Fluorescence-activated cell sorting profiles and gates used to collect H1.2-HaloTag-TMR positives are also indicated in fig. S2A. The number of cells collected per total number of cells analyzed is also indicated for each run. Cells were collected into 2 ml of ice-cooled collection medium [Dulbecco’s modified Eagle’s medium (DMEM) consisting of 50% fresh and 50% conditioned media (harvested from log phase growth cells and 0.2 μm filtered)]. Fetal bovine serum (FBS) and penicillin-streptomycin were added to final concentrations of 20% and 2.5 μg/ml, respectively. Cells were expanded, and colonies were isolated. Proper insertions were confirmed by PCR with the following primers: 5′-GGCCCCTGTAAAGAAGAAGG-3′ and 5′-GGACAAAATGGCTGGCTAAA-3′.

For RPE-1 cells that stably express H1.0-HaloTag from the endogenous locus, we used a similar strategy for H1.2-HaloTag HCT116 cells. Proper insertions were confirmed by PCR with the following primers: 5′-AGGAAAAGCAGCGACTCCTC-3′ and 5′-CTGCTCCCTACCACCTCTCT-3′.

To further express H2B-PAmCherry in the obtained RPE-1 cells, pPB-PGKneo-EF1α-H2B-PAmCherry-BGH-polyA (*6*) and pCMV-hyPBase (provided by the Sanger Institute with a materials transfer agreement) were cotransfected to RPE-1 cells expressing H1.2-Halo with the Effectene Transfection Reagent kit (301425; QIAGEN). The transformants were then selected using G418 (600 μg/ml).

To create HCT116 H1.2-mAC cells, HCT116 cells with CMV-OsTIR1-F74G (*64*) were cotransfected with 750 ng each of pX330 (with a target gene–specific guide sequence inserted) and the H1.2-mAC donor plasmid using the Neon electroporation transfection system (Thermo Fisher Scientific, MPK1025). The transformants were enriched using a cell sorter, and colonies were isolated. Proper insertions were confirmed by PCR with the following primers: 5′-GGCCCCTGTAAAGAAGAAGG-3′ and 5′-GGACAAAATGGCTGGCTAAA-3′. To further express H2B-Halo in the obtained HCT116 H1.2-mAC cells, pPB-CAG-IB-H2B-HaloTag (*69*) and pCMV-hyPBase were cotransfected. The transformants were then selected with blasticidin S (10 μg/ml; 029-18701; Wako; pPB-CAG-IB-H2B-HaloTag).

RPE-1 cells were cultured in DMEM (D5796; Sigma-Aldrich) supplemented with 10% FBS (F7524, Sigma-Aldrich) at 37°C under 5% CO_2_. HCT116 cells were cultured in McCoy’s 5A medium (16600-082; Gibco) supplemented with 10% FBS (F7524, Sigma-Aldrich) at 37°C under 5% CO_2_.

### Biochemical fractionation of nuclei from cells expressing H1.2-HaloTag

Nuclei were isolated from RPE-1 cells expressing endogenous H1.2-HaloTag, as described previously (*96*). Briefly, collected cells were suspended in nucleus isolation buffer [3.75 mM tris-HCl (pH 7.5), 20 mM KCl, 0.5 mM EDTA, 0.05 mM spermine, 0.125 mM spermidine, aprotinin (1 μg/ml) (T010A; TaKaRa), and 0.1 mM phenylmethylsulfonyl fluoride (P7626-1G; Sigma-Aldrich)] and centrifuged at 1936*g* for 7 min at room temperature. The cell pellets were resuspended in nucleus isolation buffer and again centrifuged at 1936*g* for 7 min at room temperature. Subsequent steps were performed at 4°C, unless otherwise noted. Cell pellets were resuspended in nucleus isolation buffer containing 0.025% Empigen (45165-50ML, Sigma-Aldrich) (nucleus isolation buffer+) and homogenized immediately with 10 downward strokes of a tight Dounce pestle (357546; Wheaton). The cell lysates were centrifuged at 4336*g* for 5 min. The nuclear pellets were washed in nucleus isolation buffer+. The nuclei were incubated on ice for 15 min in the following buffers containing various concentrations of salt: HE [10 mM Hepes-NaOH (pH 7.5), 1 mM EDTA, and 0.1 mM phenylmethylsulfonyl fluoride], HE + 100 mM NaCl, HE + 200 mM NaCl, HE + 300 mM NaCl, and HE + 500 mM. After each buffer incubation with increasing concentrations of salt, centrifugation was performed to separate the nuclear suspensions into supernatant and pellet fractions. The proteins in the supernatant fractions were precipitated by using 17% trichloroacetic acid (208-08081; Wako) and cold acetone. Both pellets were suspended in a Laemmli sample buffer and subjected to 12.5% SDS–polyacrylamide gel electrophoresis, followed by Coomassie brilliant blue (031-17922; Wako) staining and Western blotting using rabbit anti-H1.2 (19649-1-AP; Proteintech) and rabbit anti-HaloTag (G9281; Promega) antibodies to confirm H1.2-HaloTag expression.

### Bulk HaloTag labeling and fixed-cell imaging

H2B-HaloTag– or H1.2/H1.0-HaloTag–expressing cells were grown on poly-l-lysine–coated (P1524-500MG, Sigma-Aldrich) coverslips (C018001, Matsunami) and labeled with 40 nM HaloTag TMR ligand (G8251, Promega) overnight. The cells were fixed with 1.85% FA (064-00406, Wako) at room temperature for 15 min, permeabilized with 0.5% Triton X-100 (T-9284, Sigma-Aldrich) for 5 min, and stained with DAPI (0.5 μg/ml) (10236276001, Roche) for 5 min, followed by PPDI [20 mM Hepes (pH 7.4), 1 mM MgCl_2_, 100 mM KCl, 78% glycerol, and paraphenylene diamine (1 mg/ml) (695106-1G, Sigma-Aldrich)] mounting. *z*-Stack images with a 0.2-μm thickness were acquired with a DeltaVision system (Applied Precision) with a UPlanApo 60× 1.40–numerical aperture (NA) objective lens (Olympus). The *z*-stack images were deconvolved with softWoRx (Applied Precision) and presented as maximum intensity projections of five sections.

### HaloTag labeling for single-molecule imaging

Cells stably expressing H1.2/H1.0-Halo or H2B-Halo were cultured on poly-l-lysine–coated glass-based dishes (3970-035, Iwaki). HaloTag-tagged molecules were fluorescently labeled with 30 to 50 pM HaloTag TMR ligand (G8251, Promega), 40 nM PA-JF646 HaloTag (for dual-color imaging), or 20 nM PA-JF549 HaloTag (for metaphase imaging) (provided by the Lavis lab at the Janelia Research Campus, VA) for 20 min; washed three times with Hanks’ balanced salt solution (HBSS) buffer (H1387; Sigma-Aldrich); and then imaged in live cells in phenol red–free DMEM with 10% FBS.

### Single-molecule imaging microscopy

Single molecules were observed using an inverted Nikon Eclipse Ti microscope with a 100-mW Sapphire 561-nm laser (Sapphire-561-100CW CDRH, Coherent) and the scientific complementary metal-oxide semiconductor (sCMOS) ORCA-Fusion BT camera (C15440-20UP, Hamamatsu Photonics). Fluorescently labeled molecules in living cells were excited by the 488-, 561-, or 640-nm lasers through an objective lens (100× Apo TIRF, NA 1.49; Nikon), and their emissions were detected. An oblique illumination with a TIRF unit (TI-TIRF-E, Nikon) was used to excite labeled molecules within a thin area in the cell nucleus and reduce the background noise. Hundreds of sequential image frames were acquired using MetaMorph (Molecular Devices) or NIS Elements (Nikon Solutions) at 50 ms per frame under continuous illumination. To maintain cell culture conditions (37°C, 5% CO_2_, and humidity) under the microscope, a live-cell chamber with a digital gas mixer and a warming box (Tokai Hit) were used.

### Single-molecule tracking and analysis

Sequential microscopy images were converted to 16-bit grayscale, and the background signals were subtracted with the rolling ball background subtraction (radius, 50 pixels). The nuclear regions in the images were manually extracted. Single-molecule detection and tracking were performed using the Fiji package [TrackMate (*72*)] with the following parameters (detector: LoGdetector; object diameter: 7 pixels; quality threshold: 2; subpixel localization, initial thresholding: 7-60 (variable depending on the experiment); tracker: LAP Tracker; linking distance: 10 or 2 pixels). For single-nucleosome movement analysis, the displacement distribution and the MSD of the fluorescent dots were calculated on the basis of their trajectory using a Python program. The originally calculated MSD was in 2D. To obtain the 3D value, the 2D value was multiplied by 1.5 (4 to 6 Dt). Graphs of the obtained single-nucleosome MSD between various conditions were obtained using R or Python. To ascertain the position determination accuracy of the molecules labeled with TMR in FA-fixed cells, distributions of nucleosome displacements from the centroid of the trajectory in the *x* and *y* planes in the 50-ms interval were fitted to Gaussian functions. The mean of SD was used for position determination accuracy.

### Motion classification by vbSPT

Trajectories longer than four frames were used as input to the vbSPT algorithm (*73*) in MATLAB by imposing a maximum number of states equal to 3 or 2. The algorithm provided the transition rates between these states. For accurate estimation, the diffusion coefficient (*D*) for each state was calculated from the 10 ms per frame data. Also, on the basis of the predicted model, steps in trajectories were annotated into each state. The annotation was used for splitting trajectories that only contained each state.

### Dual-color imaging (PALM + single-molecule imaging)

Single molecules were observed using an inverted Nikon Eclipse Ti microscope with an ILE laser unit (ANDOR) and the sCMOS ORCA-Fusion BT camera. Fluorescent molecules in living cells were excited by the 561- or 640-nm laser through an objective lens (100× Apo TIRF, NA 1.49; Nikon) and detected. A weak (0.1%) 405-nm laser was also used for photoactivation of PAmCherry and PA-JF646. An oblique illumination system with a TIRF unit (TI2-LA-TIRF-E, Nikon) was used to excite labeled molecules within a limited thin area in the cell nucleus and reduce the background noise. Sequential 1000 images were acquired using NIS Elements (Nikon solutions) at a frame rate of 50 ms under continuous illumination. To maintain cell culture conditions (37°C, 5% CO_2_, and humidity) under the microscope, a live-cell chamber with a digital gas mixer and a warming box (Tokai Hit) were used. Beam splitter W-VIEW GEMINI (Hamamatsu Photonics) was used with filters. Chromatic shifts of channels were aligned manually with quad-fluorescent beads.

### Dual-color imaging (single-molecule imaging + mitotic chromosome)

H1.2-Halo was labeled with PA-JF549 HaloTag. To stain mitotic chromosomes in live cells, 100 nM SiR-Hoechst (SiR-DNA, no. CY-SC007; Spirochrome) was added to the medium. Fluorescent molecules in living cells were excited by the 561- or 640-nm laser. Beam splitter W-VIEW GEMINI (Hamamatsu Photonics) was used with filters. Cells were asynchronous.

### Colocalization analysis of chromatin domain and H1

PALM images were constructed using the Fiji package ThunderSTORM (*97*) with ×2 magnification on the basis of sequential images of PAmCherry, resulting in 32.5 nm by 32.5 nm for a pixel and 740 px–by–740 px image. In the middle of the nucleus, 220 px by 220 px was cropped for this analysis to avoid the nuclear periphery. All pixels were classified into four classes by quad percentile on the basis of the intensity distribution. From classified trajectories by vbSPT, each middle point of a step belonged to a pixel, so the total number of steps could be calculated for each class. Enrichment for each class (number of steps with the state included in the class/number of all steps with the state) was calculated for single-cell data, and then data from more than five cells were averaged for results. If steps were distributed randomly, each class should contain 25% of steps so that enrichment is colored with 0.25 as a border. A PALM image and trajectories were visualized by a Python script.

### PALM imaging for fixed cells

Cells stably expressing H2B-Halo were cultured on poly-l-lysine–coated glass-based dishes (3970-035, Iwaki). HaloTag-tagged molecules were fluorescently labeled with 200 nM HaloTag PA-JF646 ligand (provided by the Lavis lab, Janelia Research Campus) for 20 min. Cells were washed three times with HBSS buffer (H1387; Sigma-Aldrich) and then incubated with phenol-free McCoy’s 5A medium (49431-32, KANTO CHEMICAL) with 10% FBS and 0.01% dimethyl sulfoxide (DMSO) or 10% FBS and 1 μM 5-Ph-IAA for 3 hours. Cells were fixed with 3.7% FA (064-00406, Wako) in Opti-MEM (31985062, Gibco) at room temperature for 15 min, washed three times with 1× PBS, and observed in phenol red–free McCoy’s 5A medium with 10% FBS. Here, PA-JF646 was illuminated with stronger (640 nm) and weaker (405 nm) lasers. The H1.2-mClover signal was also detected using a 488-nm laser.

### Clustering analyses of nucleosomes in PALM images

The methods for clustering analyses of nucleosomes in PALM images were described previously (*6*). Ripley’s *K* function is given by

$$K\left(r\right)=\left(\frac{S}{N-1}\right)\left[\frac{1}{N}\sum_{i=1}^{N}\sum_{i\neq j}\delta \left(r-r_{i,j}\right)\right]$$

where (*N* − 1)/*S* is the average particle density of area *S*, and *N* is the total number of particles contained in the area. The delta function is given by

$$\delta \left(r-r_{i,j}\right)=\left\{ \begin{array}{cc} 1, & r_{i,j}\leq r\\ 0, & r_{i,j}>r \end{array}\right.$$

where *r*_*i*,*j*_ is the distance between *r_i_* and *r_j_*.

The *L* function is given by

$$L\left(r\right)=\sqrt{\frac{K\left(r\right)}{\pi }}$$

The area *S* of the total nuclear region was estimated using the Fiji plug-in Trainable Weka Segmentation, and the area of the whole region was measured by Analyze Particles (ImageJ).

### Expression of wild-type H1.2 and mutated H1.2 in HCT116 cells

Either wild-type H1.2-HaloTag (H1.2-WT) or mutated H1.2-HaloTag (H1.2-Khalf) was transiently expressed using the Tet-On inducible system. Inducible vectors of H1.2-WT or H1.2-Khalf were transfected into HCT116 cells with H1.2-mAC using the Neon electroporation transfection system and seeded on a glass-bottom dish. Two days after transfection, cells were treated with 1 μM 5-Ph-IAA and doxycycline (1 μg/ml) for 6 hours to deplete endogenous H1.2, and ectopic expression of H1.2-WT or H1.2-Khalf was induced. Then, HaloTag-tagged H1.2-WT or H1.2-Khalf was fluorescently labeled with 10 pM JFX650 HaloTag ligand (HT1070, Promega) and 100 nM HaloTag TMR ligand for 20 min. The cells were washed three times with HBSS buffer (H1387; Sigma-Aldrich) and then observed in phenol red–free McCoy’s 5A medium with 10% FBS, 1 μM 5-Ph-IAA, and doxycycline (1 μg/ml). Red fluorescence from TMR was used for searching cells that expressed H1.2-WT or H1.2-Khalf, and far-red fluorescence from JFX650 was used for single-molecule tracking.

### RL algorithm

We used the RL algorithm to derive a smooth distribution curve from the sparse and noisy histogram of the single-cell molecule trajectory data (*75*). The single-cell distributions of the 2D MSD, denoted by M, are shown in Fig. 3B and fig. S5F. These distributions were obtained by numerically iterating the RL calculation. To start, we sampled the 2D position ${\vec{r}}_{i}\left(t\right)$ of the ith molecule of the αth cell (i∈α) at time t. We then calculated the self-part of the van Hove correlation function

$$G_{s}\left(r,\Delta t;\alpha \right)={\langle {\left[\delta \left(r-|{\vec{r}}_{i}\left(t_{0}+\Delta t\right)-{\vec{r}}_{i}\left(t_{0}\right)|\right)\right]}_{t_{0}}\rangle }_{i\in \alpha }$$

where δ(⋯) represents Dirac’s delta function, ${\left[\cdots \right]}_{t_{0}}$ denotes the average over different values of $t_{0}$, and ${\langle \cdots \rangle }_{i\in \alpha }$ indicates the average over the molecules i belonging to the cell α. The iteration started from an initial estimate for P(M,Δt;α) as $P^{0}\left(M,\Delta t;\alpha \right)=\text{exp}\left(-M/M_{0}\right)/M_{0}$, where the convergent results were not sensitive to the choice of the $M_{0}$ value. The RL calculation was iterated using

$$P^{n+1}\left(M,\Delta t;\alpha \right)=P^{n}\left(M,\Delta t;\alpha \right)\int 2\pi r\frac{q\left(r,M\right)G_{s}\left(r,\Delta t;\alpha \right)}{G_{s}^{n}\left(r,\Delta t;\alpha \right)}\mathrm{dr}$$

under constraints $P^{n+1}\left(M;\alpha \right)\geq 0$ and $\int {\mathrm{dMP}}^{n+1}\left(M;\alpha \right)=1$. Here, $q\left(r,M\right)=\text{exp}\left(-r^{2}/M\right)/\left(\pi M\right)$ and $G_{s}^{n}\left(r,\Delta t;\alpha \right)=\int \mathrm{dMq}\left(r,M\right)P^{n}\left(M;\alpha \right)$. The process was continued until the difference between $P^{n+1}\left(M;\alpha \right)$ and $P^{n}\left(M;\alpha \right)$ became sufficiently small, resulting in the convergent distribution $P\left(M,\Delta t;\alpha \right)=P^{n+1}\left(M,\Delta t;\alpha \right)$. While the quantitative features of P(M,Δt;α) vary from cell to cell, the qualitative features—including the number of peaks and the rough difference among peak positions—remain consistent across different cells. Therefore, distributions P(M,Δt;α) depicted for an example cell α in Fig. 3B and fig. S5F reflect the essential characteristics common to the n=40 cells examined.

### Mean-field theory of H1 and nucleosomes

The multiscale modeling of interactions between H1 molecules and nucleosomes (Figs. 1 and 5) demonstrated that H1 can interact with multiple nucleosomes in a short period, leading to weak multivalent interactions. We anticipate that these interactions are associated with both a decrease in energy and increase in entropy. This entropy gain arises because a transiently binding dynamic H1 molecule can adopt multiple interaction states with neighboring nucleosomes. This variability in interactions offsets the decrease in entropy that results from reduced translational movement. To investigate whether these multivalent interactions and the resulting entropy gain stabilize the dynamic H1 molecules, we examined a mean-field model similar to the lattice polymer model of Flory and Huggins (fig. S9A) (*83*). In this model, we assume that chromatin chains, composed of K nucleosomes, are placed on the lattice with $N_{0}$ grid points. Each grid point can either be occupied by a nucleosome or be left unoccupied, resulting in a total of N nucleosomes; therefore, the nucleosome density is given by $N/N_{0}$. H1 is modeled to jump over the lattice sites with a chemical potential μ when it is not bound to a nucleosome. Each nucleosome can either bind one H1 molecule or remain unbound. When H1 binds at the most stable dyad position of a nucleosome, H1 is stabilized by an energy of −(ϵ+δ) with ϵ>0 and δ>0. If H1 dynamically binds near the dyad, it has a higher binding energy of −ϵ<0. We assume that such dynamically bound H1 can interact with nucleosomes at neighboring lattice sites, with an interaction energy of −J<0. Then, the mean-field energy can be expressed as

$$E\left(m,n;N\right)=-\mathrm{Jm}\left[1-{\left(1-\frac{N}{N_{0}}\right)}^{z}\right]-\left(\mu +\epsilon \right)m-\left(\mu +\epsilon +\delta \right)n$$

where m represents the number of H1 molecules with dynamic binding, n is the number of H1 molecules with stable binding, and z is the number of neighboring lattice sites. The first term on the right-hand side of this equation accounts for the multivalent interactions between H1 and nucleosomes, representing the nonlinear dependence of energy on $N/N_{0}$. Entropy is derived from the number of states, estimated as

$$\Omega \left(m,n;N\right)=\Omega_{\text{nucl}}\left(N\right)\Omega_{\text{chain}}\left(N\right)\Omega_{H1}\left(m,n;N\right)\Omega_{\text{multivalent}}\left(m;N\right)$$

Here, $\Omega_{\text{nucl}}\left(N\right)=\frac{N_{0}!}{\left(N_{0}-N\right)!N!}$ shows the effects of nucleosome motion, $\Omega_{\text{chain}}\left(N\right)={\left(\frac{N}{N_{0}}\right)}^{N-N/K}$ accounts for the connection in the chromatin chain, $\Omega_{H1}\left(m,n;N\right)=\frac{N!}{n!m!\left(N-n-m\right)!}$ shows the distribution of H1 bound to nucleosomes, and $\Omega_{\text{multivalent}}\left(m;N\right)={\left(\frac{\mathrm{zN}}{N_{0}}\right)}^{m}$ represents the fluctuation in multivalent interactions between the mobile H1 and neighbor nucleosomes. The last term reflects the entropy associated with the multivalent nature of transient H1 binding. Given that each mobile H1 transiently bound to a particular nucleosome can choose a binding partner from an average of $\frac{\mathrm{zN}}{N_{0}}$ neighboring nucleosomes, the mean number of states that m mobile H1 can adopt is $\Omega_{\text{multivalent}}\left(m;N\right)={\left(\frac{\mathrm{zN}}{N_{0}}\right)}^{m}$. This captures the combinatorial variability of H1-nucleosome interactions arising from the local chromatin environment. Entropy $S=k_{B}\text{logΩ}$ with $k_{B}$ being the Boltzmann factor is

$$S\left(m,n;N\right)=S_{\text{nucl}-\text{chain}}\left(N\right)+S_{H1}\left(m,n;N\right)+S_{\text{multivalent}}\left(m;N\right)$$

with $S_{\text{nucl}-\text{chain}}\left(N\right)=k_{B}\text{log}\left(\Omega_{\text{nucl}}\left(N\right)\Omega_{\text{chain}}\left(N\right)\right)$, $S_{H1}\left(m,n;N\right)=k_{B}\text{log}\Omega_{H1}\left(m,n;N\right)$, and $S_{\text{multivalent}}\left(m;N\right)=k_{B}\text{log}\Omega_{\text{multivalent}}\left(m;N\right)$. Neglecting the higher-order terms of $1/N_{0}$, each component of entropy is

$$S_{\text{nucl}-\text{chain}}\left(N\right)=-k_{B}\left[N_{0}\left(1-\frac{N}{N_{0}}\right)\text{log}\left(1-\frac{N}{N_{0}}\right)+\frac{N}{K}\text{log}\frac{N}{N_{0}}\right]$$

$$S_{H1}\left(m,n;N\right)=-k_{B}\left[m\text{log}\frac{m}{N_{0}}+n\text{log}\frac{n}{N_{0}}+\left(N-m-n\right)\text{log}\left(\frac{N-m-n}{N_{0}}\right)\right]$$

$$S_{\text{multivalent}}\left(m;N\right)=-k_{B}\left[-m\text{log}\frac{\mathrm{zN}}{N_{0}}\right]$$

Here, $S_{\text{multivalent}}\left(m;N\right)$ represents the entropy effect of multivalent interactions of dynamic H1. Then, the free energy per lattice site at temperature T is given by

$$f\left(m,n;N\right)=\frac{1}{N_{0}}\left[E\left(m,n;N\right)-\mathrm{TS}\left(m,n;N\right)\right]$$

In fig. S9B, f(m,n;N) is plotted on a 2D plane representing the ratio of H1 on the nucleosome, (n+m)/N, and the ratio of H1 with dynamic binding, m/(n+m). To represent the stable binding of H1 at the dyad position of nucleosomes, we assume that δ>ϵ≈μ≈J>0. The parameters used for the results in fig. S9 were chosen as K=1000, z=6, $\delta /k_{B}T=1.5$, and $\epsilon /k_{B}T=\mu /k_{B}T=J/k_{B}T=1$.
