# Supplementary material for: Linker histone H1 functions as a liquid-like glue to organize chromatin in living human cells
Source: Sci Adv. 2026 Apr 8;12(15):eaec9801. doi: 10.1126/sciadv.aec9801 (PMC13060598; doi:10.1126/sciadv.aec9801)
Supplement: Supplementary file 1 — Figs. S1 to S9 Legends for movies S1 to S7 [file sciadv.aec9801_sm.pdf]

Science Advances
AAAS

Supplementary Materials for

Linker histone H1 functions as a liquid-like glue to organize chromatin in living human cells

Masa A. Shimazoe *et al.*

Corresponding author: Kazuhiro Maeshima, kmaeshim@nig.ac.jp; Rosana Collepardo-Guevara, rc597@cam.ac.uk

Sci. Adv. **12**, eaec9801 (2026)
DOI: 10.1126/sciadv.aec9801

The PDF file includes:

Figs. S1 to S9
Legends for movies S1 to S7

Other Supplementary Material for this manuscript includes the following:

Movies S1 to S7

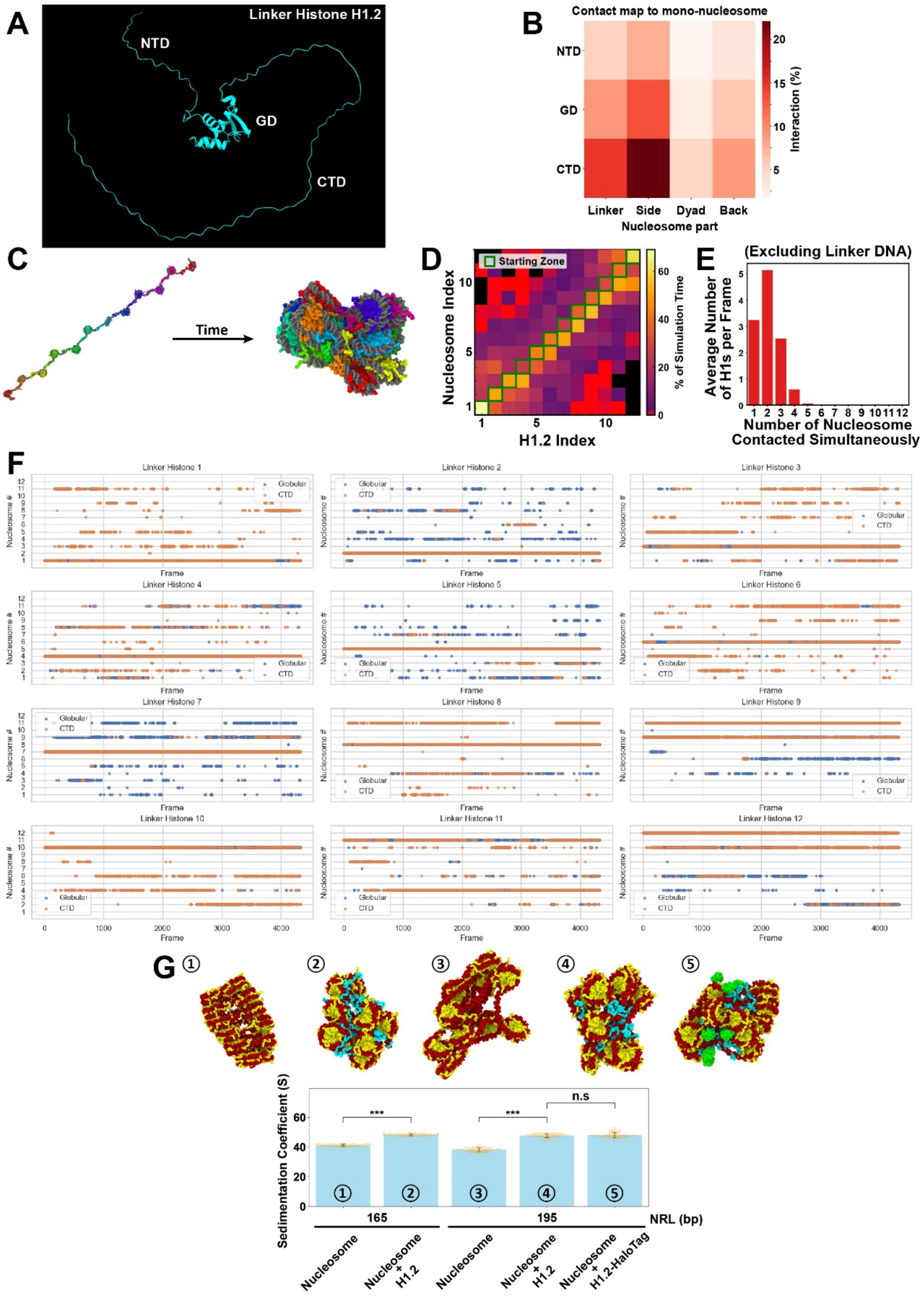

Fig. S1. Models of a linker histone H1 and a 12-mer nucleosome array.

(A) Structure of human linker histone H1.2 predicted by AlphaFold3: unstructured N-terminal domain (NTD), globular domain (GD), and a long, intrinsically disordered C-terminal domain

(CTD). **(B)** Region-specific contact map between a H1.2 molecule and a mono-nucleosome. H1.2 (y-axis): NTD, GD, CTD regions; Nucleosome (x-axis): Linker, Side, Dyad, and Back regions. **(C)** Initial configuration (left) and final configuration (right) of a 12-mer nucleosome array (195 bp NRL) with H1.2 (Nucleosome:H1 = 1:1). Originally, each H1.2 was attached to one nucleosome on a linear array, then H1.2 translocated to other nucleosomes over time, twisting and condensing the array. **(D)** Contact map between twelve nucleosomes and twelve H1.2 on the array (195 bp NRL). Starting zones on the diagonal (square edge in green) indicate where H1.2 is located at the beginning of the simulation. Note that H1.2 often left the original nucleosome and translocated among several nucleosomes. **(E)** Histogram of the number of nucleosomes contacted simultaneously by an H1.2. Only core nucleosomal DNA was taken into account. **(F)** Most-contacted nucleosome (excluding linker DNA) in an unbiased MD simulation of a 12-mer nucleosome array with 195 bp NRL. For each linker histone, the nucleosome with the highest average number of contacts per frame is shown separately for the GD and CTD. **(G)** Examples of nucleosome array configurations and their corresponding sedimentation coefficients (S) (± standard deviation, SD) with different conditions. With H1.2, the nucleosome arrays show a significantly higher S, indicating greater compaction. Similar trends were observed for different NRLs or with a H1.2-HaloTag (green sphere). The Wilcoxon rank-sum test was used to determine P values and corrected by the Benjamini-Hochberg method. ***$P < 0.0001$ for nucleosomes with 165 bp NRL versus nucleosomes with 165 bp NRL + H1.2 ($P = 4.2 \times 10^{-34}$), nucleosomes with 195 bp NRL versus nucleosomes with 195 bp NRL + H1.2 ($P = 4.2 \times 10^{-34}$). Not significant (N.S.) for nucleosomes with 195 bp NRL + H1.2 versus nucleosomes with 195 bp NRL + H1.2-Halo ($P = 0.89$).

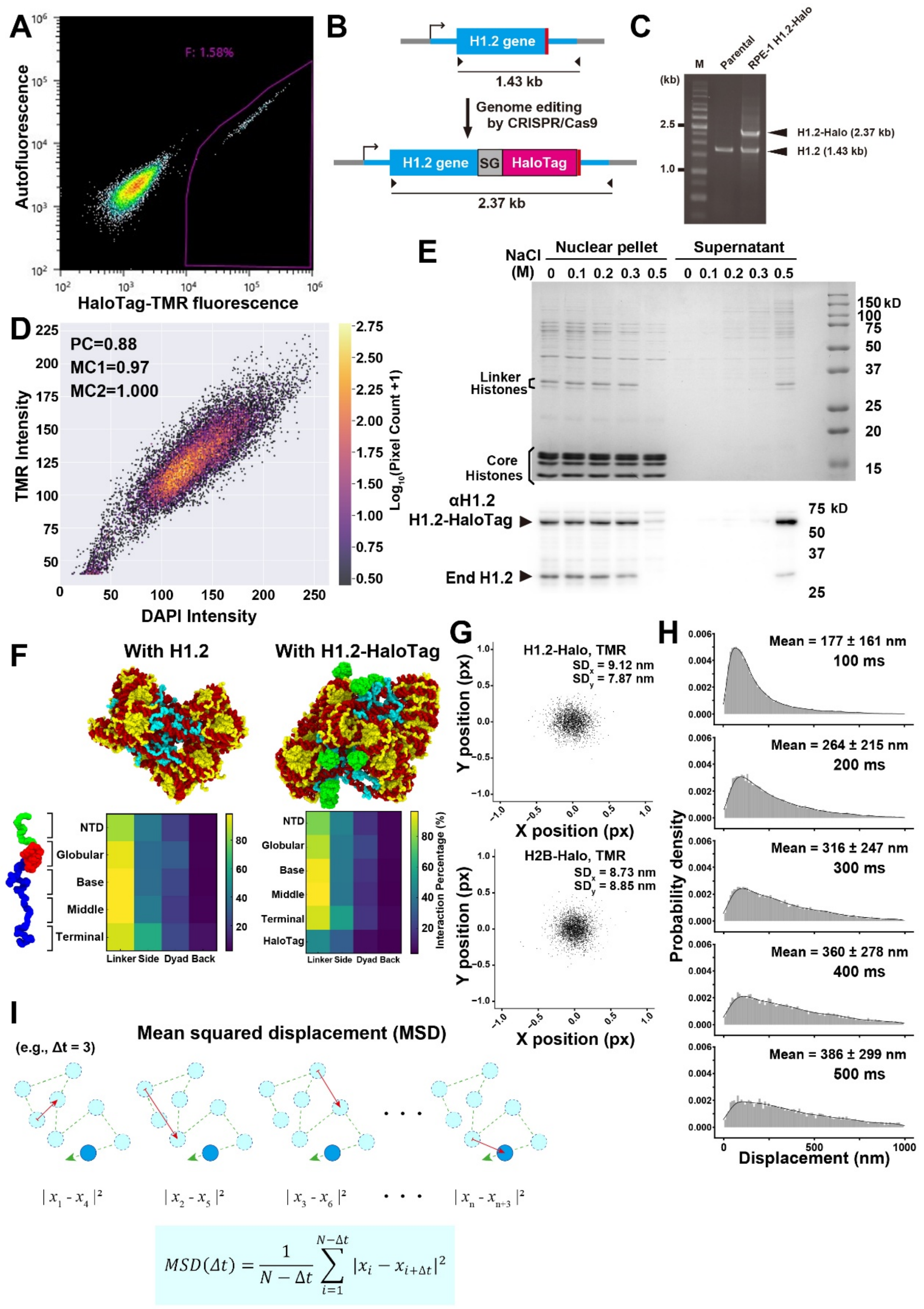

Fig. S2. Generation of RPE-1 cells expressing the H1.2-HaloTag and characterization of H1.2-Halo behavior.

(A) Intensity scatter plot of FACS for sorted RPE-1 cells expressing H1.2-HaloTag (H1.2-Halo). The gate used to collect Halo-TMR positive cells is shown with a pink line. **(B)** Scheme for the

original and HaloTag-inserted H1.2 gene loci and expected fragments amplified by PCR with the indicated primer set. **(C)** Validation for proper mono-allelic insertion of the HaloTag in RPE-1 genomic DNA by PCR: Parental (left) and H1.2-Halo (right) RPE-1 cells. HaloTag was inserted into the heterozygous H1.2 gene loci. **(D)** Pixel intensity correlation of DAPI and TMR staining in Fig. 2B. PC, Pearson correlation coefficient; MC1/MC2, Manders correlation coefficients. **(E)** Stepwise-salt washing of nuclei isolated from the H1.2-Halo expressing RPE-1 cells. The nuclei were washed with buffers containing increasing concentrations of NaCl. The resultant nuclear pellets (left) and supernatants (right) were analyzed by SDS-PAGE, and subsequently stained with Coomassie brilliant blue (top) or immunoblotted with anti-H1.2 (bottom). Positions of core histones and linker histone H1 are indicated on the Coomassie brilliant blue stained gel. Note that H1.2 and H1.2-Halo dissociate from chromatin with 0.5 M NaCl and were detected in the supernatant fraction, suggesting that H1.2-Halo interacts with chromatin like endogenous H1.2. **(F)** Representative configuration and region-specific contact map of 12-mer nucleosome array with H1.2 or H1.2-Halo. Note that both conditions showed compact, irregular clusters and similar region-specific contact maps. **(G)** Scatter plot of H2B-Halo-TMR (n = 214) and H1.2-Halo-TMR (n = 155) dots in formaldehyde (FA)-fixed cell to ascertain the position determination accuracy. Standard deviations on the x-axis and y-axis are shown as SD_x and SD_y. **(H)** Displacement distribution histograms (n = 40 cells) for 100, 200, 300, 400, and 500 ms. Means ± SD of displacement are at the top of each. **(I)** Calculation of mean squared displacement (MSD).

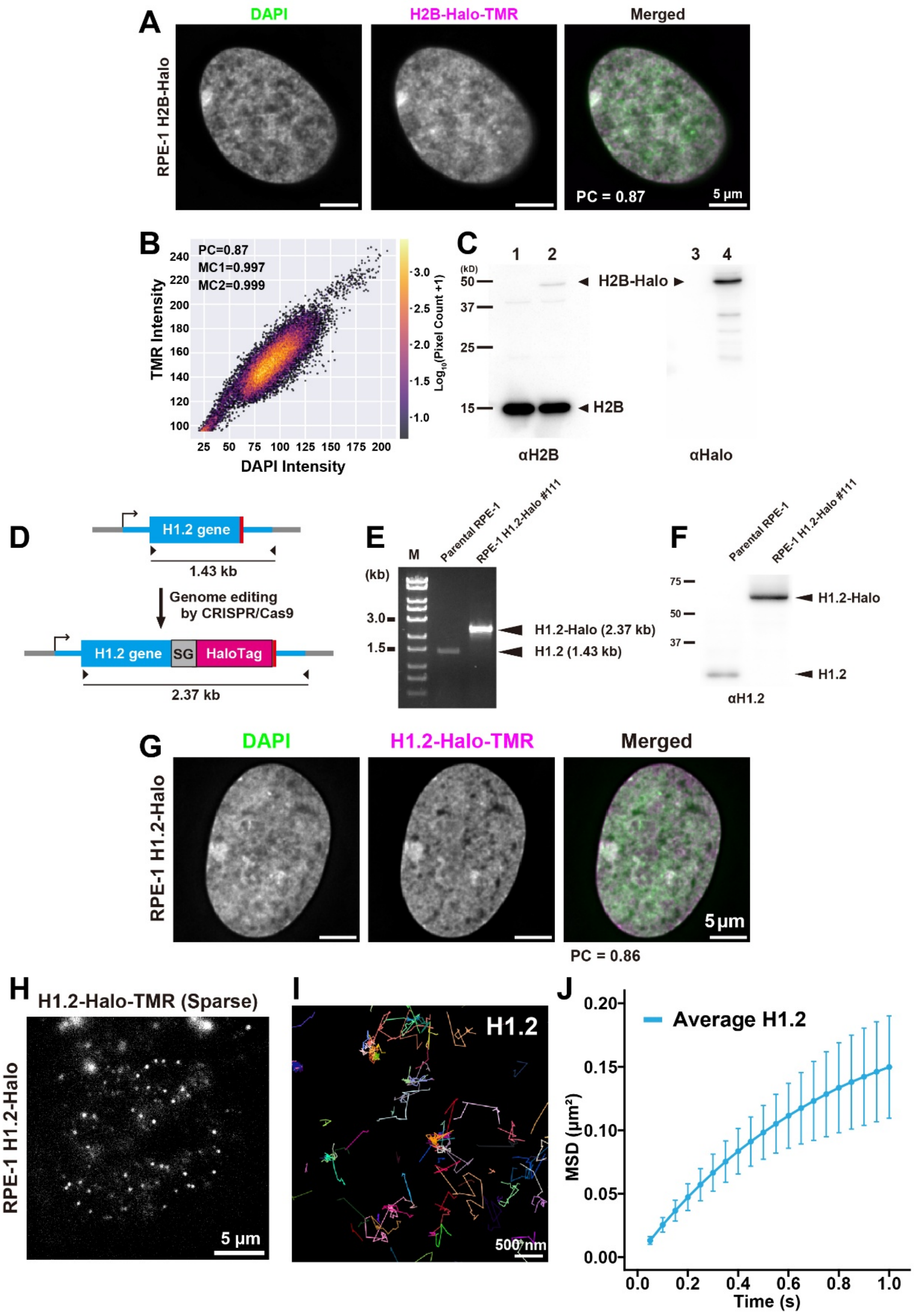

Fig. S3. Generation of RPE-1 cells ectopically expressing H2B-Halo and of RPE-1 cells with bi-allelic HaloTag knock-in at the H1.2 locus.

(A) Representative images of RPE-1 cells ectopically expressing H2B-Halo. The localization of H2B-Halo-TMR is similar to that of DAPI. **(B)** Pixel intensity correlation of the DAPI and TMR

images in Fig. S3A. PC, Pearson correlation coefficient; MC1/MC2, Manders correlation coefficient. **(C)** Western blotting of parental RPE-1 (lanes 1, 3) and RPE-1 expressing H2B-Halo (lanes 2, 4) using anti-H2B antibody (lanes 1, 2) and anti-HaloTag antibody (lanes 3, 4). **(D)** Scheme for the parental and HaloTag-inserted H1.2 gene loci and expected fragments amplified by PCR with the indicated primer set. **(E)** Validation for bi-allelic insertion of the HaloTag in RPE-1 genomic DNA by PCR: Parental (left) and H1.2-Halo (right) RPE-1 cells. **(F)** Western blotting of parental RPE-1 (left) and RPE-1 expressing H1.2-Halo (right) using an anti-H1.2 antibody. Note that all H1.2 is tagged by HaloTag. **(G)** Representative images of RPE-1 cells expressing H1.2-HaloTag via bi-allelic HaloTag insertion. The localization of H1.2-Halo-TMR is similar to that of DAPI. **(H)** A representative single-molecule image of H1.2-Halo-TMR in the generated RPE-1 cells. Each white dot represents a single molecule of H1.2-Halo. **(I)** Representative trajectories of single nucleosomes labeled with H1.2-Halo-TMR (right) acquired at 50 ms/frame. **(J)** Mean squared displacement (MSD) plots (± SD among cells) of single H1.2 molecules (blue) in living RPE-1 cells in a tracking time range from 0.05 to 1 s (n = 30 cells).

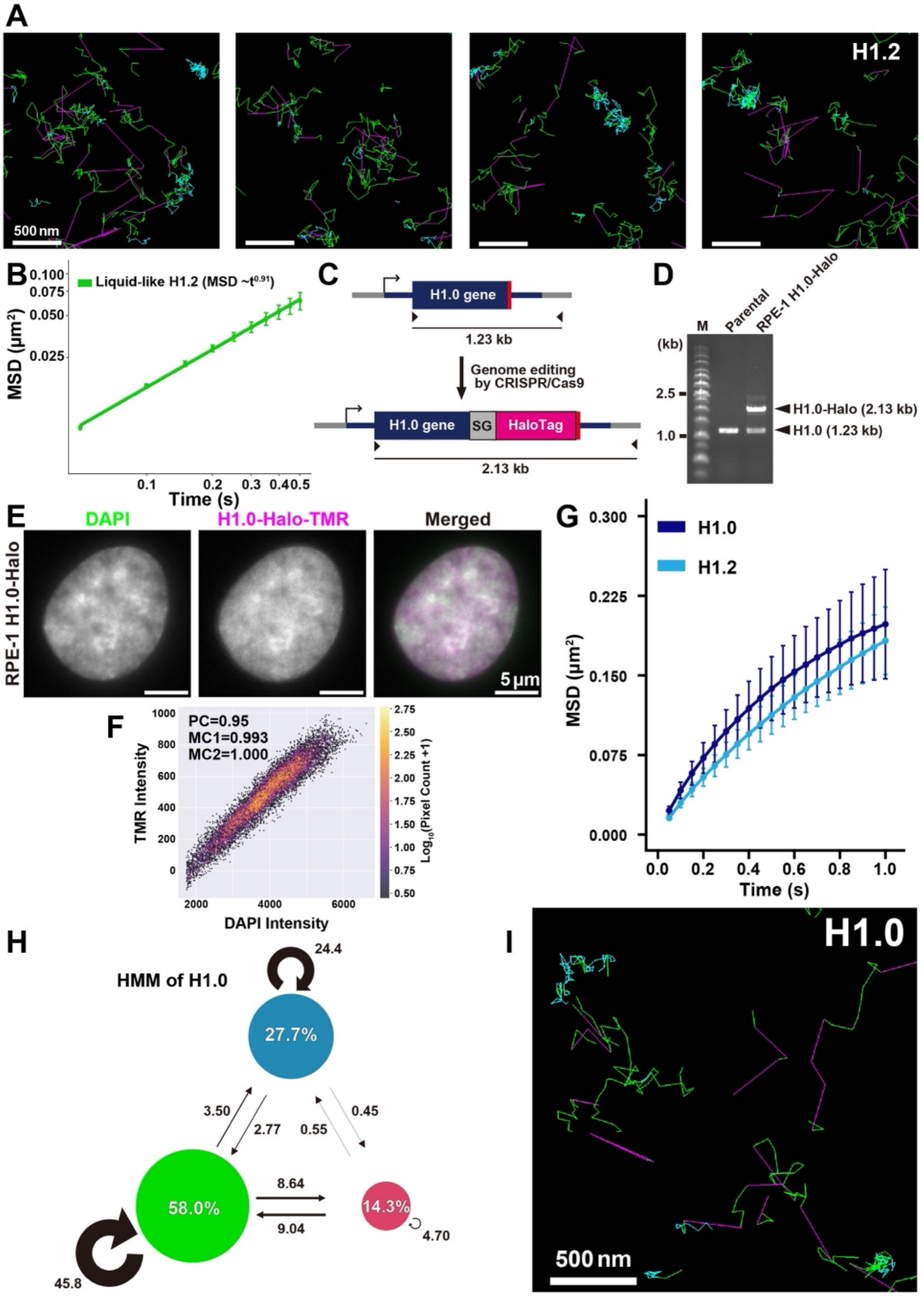

Fig. S4. Further trajectory analysis of H1.2, creation of RPE-1 cells expressing H1.0-Halo, and single-H1.0 imaging.

(A) Examples of classified trajectories of H1.2. **(B)** The log-log plot of MSDs from the plot of "liquid-like H1". The plot was fitted linearly. The calculated anomalous exponent is also shown. **(C)** Schematic of CRISPR/Cas9-mediated genome editing for inserting the HaloTag at the C-

terminus of the H1.0 gene locus. SG, linker (GGGGS x2). Expected PCR fragment lengths for original and HaloTag-inserted H1.0 gene loci are shown. **(D)** Validation for proper insertion of HaloTag in RPE-1 genomic DNA by PCR: Parental (left) and H1.0-Halo (right) RPE-1 cells. HaloTag was inserted into the heterozygous H1.0 gene locus. **(E)** Representative images of FA-fixed RPE-1 cells expressing H1.0-Halo. The localization of H1.0-Halo-TMR is similar to that of DAPI. **(F)** Pixel intensity correlation of DAPI and TMR images in Fig. S4E. PC, Pearson correlation coefficient; MC1/MC2, Manders correlation coefficient. **(G)** MSD plots (± SD among cells) of single H1.0 and H1.2 in living RPE-1 cells over a tracking time range from 0.05 to 1 s (n = 40 cells for each)(H1.2 data was reproduced from Fig. 2H). **(H-I)** vbSPT classification of H1.0 trajectories (n = 40 cells).

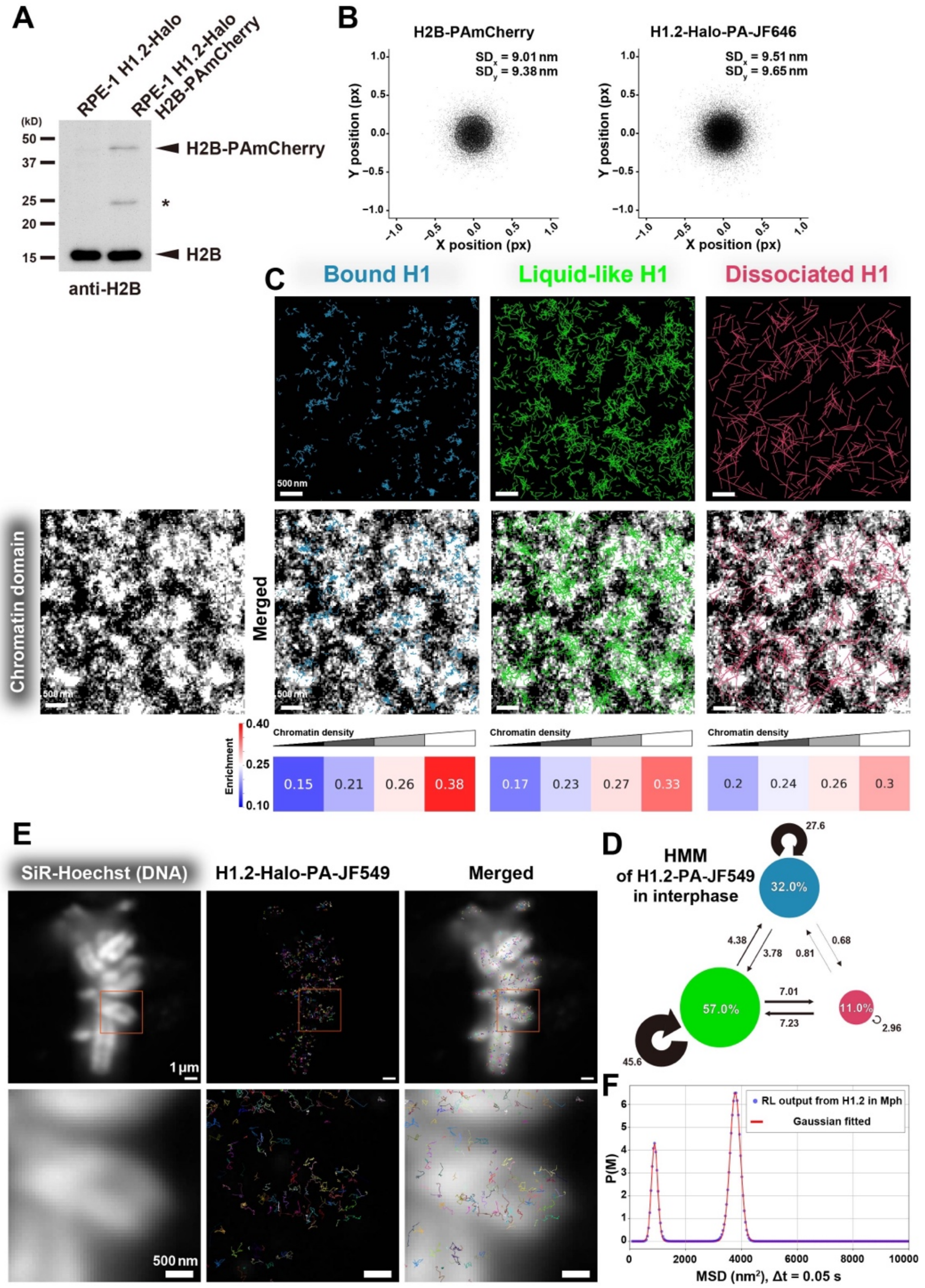

Fig. S5. Dual color imaging of H2B-PAmCherry and H1.2-Halo-PA-JF646, and H1.2 movements in metaphase chromosomes.

(A) Western blotting of parental RPE-1 and RPE-1 H1.2-Halo cells expressing H2B-PAmCherry using an anti-H2B antibody. **(B)** Scatter plots of H2B-PAmCherry (n = 8927) and H1.2-Halo-PA-JF646 (n = 5236) dots in a FA-fixed cell to ascertain the position determination accuracy. Standard deviations on the x-axis and y-axis are shown as SD_x and SD_y. **(C)** Overlap of the PALM image of nucleosomes and trajectories of each state. The distribution of trajectories in

each class is shown as enrichment values and colors. A value of 0.25 represents a random distribution of trajectories. Bound H1 and liquid-like H1 show prominent enrichment in chromatin-dense regions. Note that the behavior of the “dissociated” state was simplified due to the limited time resolution (50 ms/frame), which precludes accurate analysis of its fast and potentially transient behavior. **(D)** vbSPT results for H1.2, labeled with PA-JF549 in interphase (n = 5 cells) **(E)** Another example of the overlap of SiR-Hoechst (DNA) signal and trajectories of H1 in metaphase chromosomes. Individual trajectories are randomly colored. Bottom panels are enlarged images from squared regions in top panels. Note that H1 shows diffusive movement within mitotic chromosomes. **(F)** Two prominent peaks were obtained from the MSD data of H1.2 in metaphase chromosomes using the RL algorithm (*75*). The outputs from the RL algorithm (blue dots) and the fitted Gaussian mixture (red line) are shown.

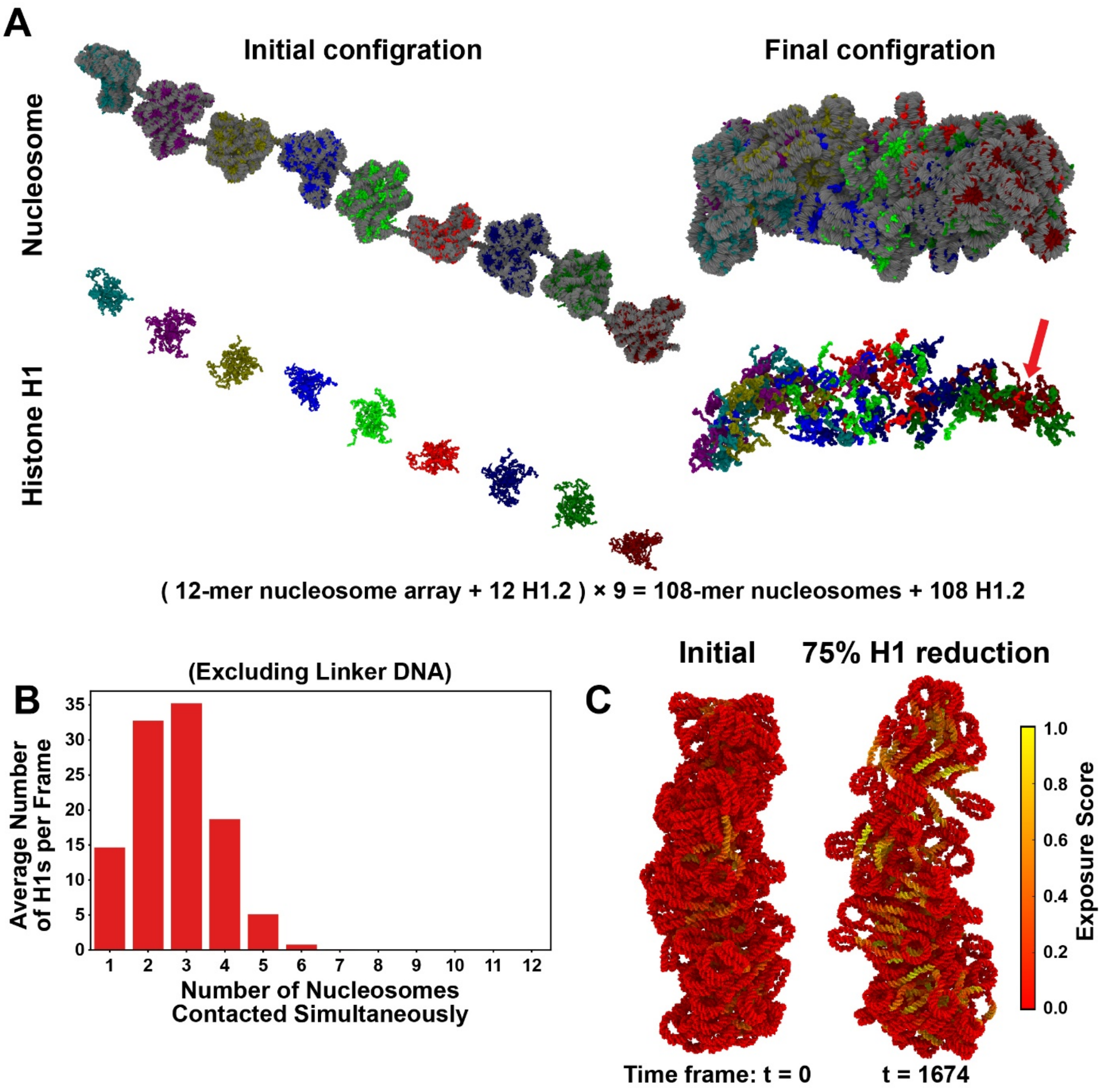

Fig. S6. H1.2 behaves like a liquid-like glue within 108 nucleosomes.
(A, left) Nine of the 12-mer nucleosome arrays (195 bp NRL) with H1.2 (one H1 per nucleosome) are connected into a fiber at the initial time point. (right) The final configuration shows an irregular cluster of 108 nucleosomes with 108 H1.2 molecules. Nucleosomes and their initially associated H1.2 are color-matched. The bottom panels display only H1. The red arrow indicates a red H1 diffuses away from its original position. Green H1, like red H1, is also dispersed at the final irregular cluster configuration, suggesting the liquid-like behavior of H1 within a dense chromatin domain. **(B)** Histogram of the number of nucleosomes contacted simultaneously by an H1.2 during the 108-nucleosome cluster simulation. Only core nucleosomal DNA was taken into account. **(C)** When 75% of H1.2 was removed from the 108-

nucleosome cluster, the cluster became decondensed and DNA was more exposed as shown with exposure score (left, initial configuration; right, final configuration after H1 reduction).

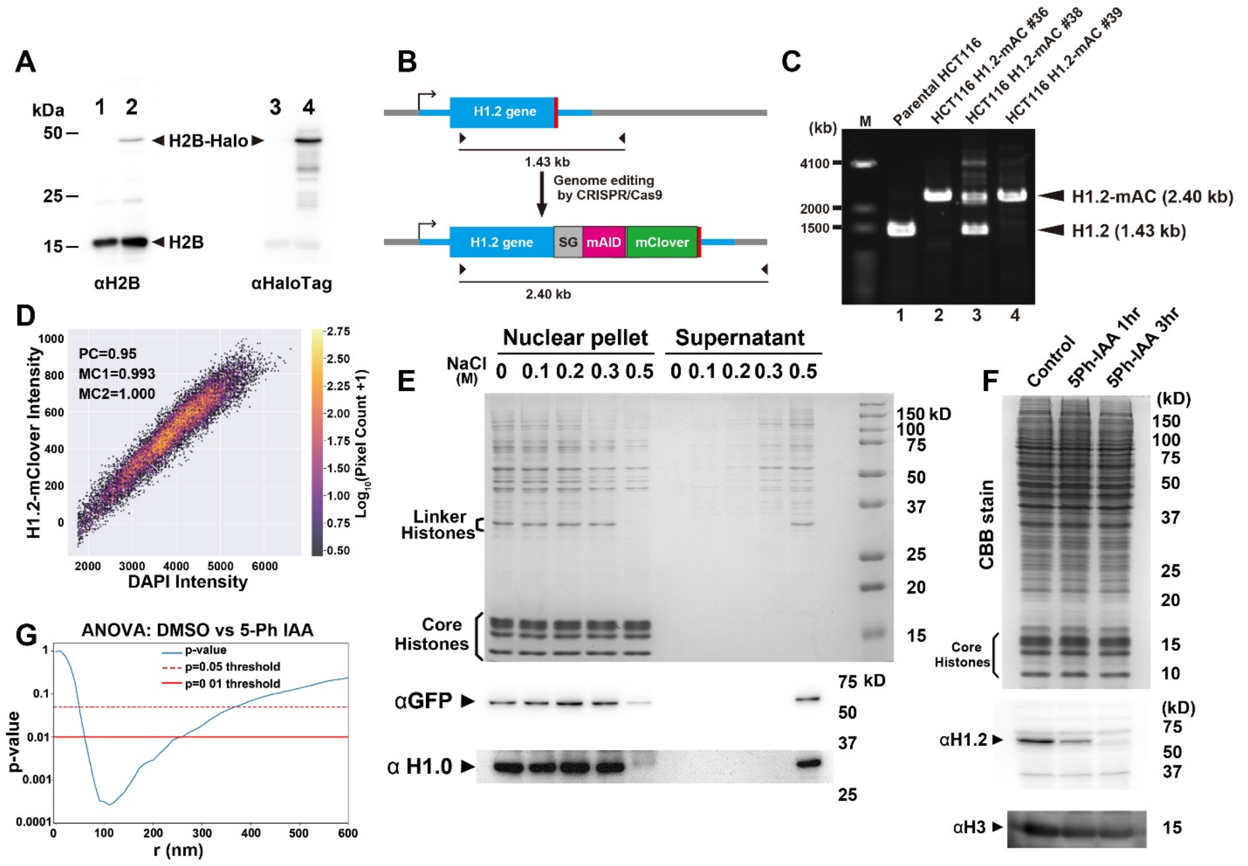

Fig. S7. Characterization of HCT116 cells expressing H1.2-mAC and H2B-Halo.
(A) Western blotting of HCT116 cells expressing H1.2-mAID-mClover (mAC)(lanes 1 and 3) and HCT116 cells expressing H1.2-mAC and H2B-Halo (lanes 2 and 4) using anti-H2B (lanes 1 and 2) and anti-HaloTag (lanes 3 and 4) antibodies. **(B)** Scheme for original and mAC-inserted H1.2 gene loci and expected fragments amplified by PCR with a primer set (arrow heads). **(C)** Validation for proper insertion of mAC by PCR in Parental (left) and H1.2-Halo (right) HCT116 cells. mAC was inserted into the homozygous H1.2 gene loci. **(D)** Pixel intensity correlation between the DAPI and H1.2-mClover images in Fig. 6C. PC, Pearson correlation coefficient; MC1/MC2, Manders correlation coefficient. **(E)** Stepwise-salt washing of nuclei isolated from HCT116 cells expressing H1.2-mAC. The isolated nuclei were washed with the buffers containing increasing concentrations of NaCl. The resultant nuclear pellets (left) and supernatants (right) were analyzed by SDS-PAGE, and subsequently stained with Coomassie brilliant blue (top) or immunoblotted with anti-GFP, anti-H1.2, or anti-H1 antibodies. Positions of core histones and linker histone H1 are indicated in the Coomassie brilliant blue stain. Note that H1.2 and H1.2-mAC dissociate from chromatin with 0.5 M NaCl and were detected in the supernatant fraction, suggesting that H1.2-mAC interacts with chromatin similar to endogenous H1.2. **(F)** Validation of rapid H1.2 depletion. Western blotting of HCT116 cells expressing H1.2-mAC using an anti-H1.2 antibody. Cells were treated with 0.01% DMSO for 3 hours (left), 1 µM 5-Ph IAA for 1 hour (center), or 1 µM 5-Ph IAA for 3 hours (right). Successful depletion of H1.2 was detected after 3 hours of 5-Ph IAA treatment. **(G)** Plot of p-values calculated for the comparison between the L-function of DMSO-treated cells (control) and 5-Ph IAA-treated cells (H1.2-depleted) in Fig. 6H. A one-way analysis of variance (ANOVA) was used to determine the

p-values. Notably, a significant reduction was observed in the range of 50 to 350 nm, corresponding to the size of chromatin domains.

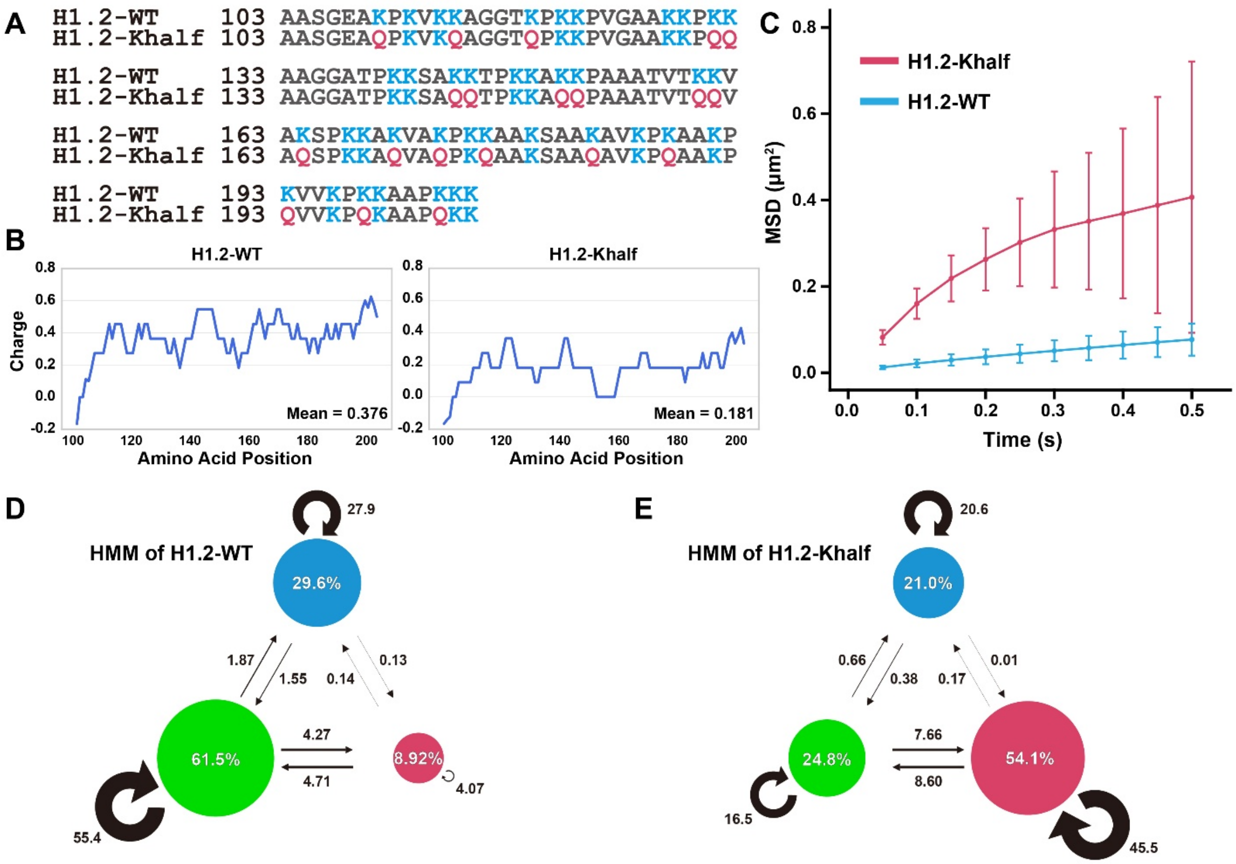

Fig. S8. Positive charge on C-terminal IDR is crucial for the liquid-like behavior of H1. **(A)** Comparison of intrinsically disordered CTD amino acid sequences between wild-type H1.2-Halo (H1.2-WT) and mutated H1.2-Halo (H1.2-Khalf). In H1.2-Khalf, half of the lysine residues were replaced with glutamine. **(B)** Charge plots of the intrinsically disordered CTD. Note that H1.2-Khalf had a lower positive charge than the wild type. **(C)** MSD plots (± SD among cells) of H1.2-WT and H1.2-Khalf in living HCT116 cells, tracked over a time range of 0.05 to 0.5 s (n = 20 cells per condition). Both molecules were sparsely labeled with JFX650 HaloTag ligand, and single molecules were tracked. **(D, E)** vbSPT results for H1.2-WT (n = 20 cells) and H1.2-Khalf (n = 20 cells), respectively. WT: $D_{Bound} = 3.26 \times 10^{-2}$ μm²/s, $D_{Liquid\text{-}like} = 1.05 \times 10^{-1}$ μm²/s, $D_{Dissociated} = 1.32$ μm²/s; Khalf: $D_{Bound} = 3.19 \times 10^{-2}$ μm²/s, $D_{Liquid\text{-}like} = 1.56 \times 10^{-1}$ μm²/s, $D_{Dissociated} = 1.63$ μm²/s. Notably, the proportion of "liquid-like H1" drastically decreased in H1.2-Khalf compared with H1.2-WT.

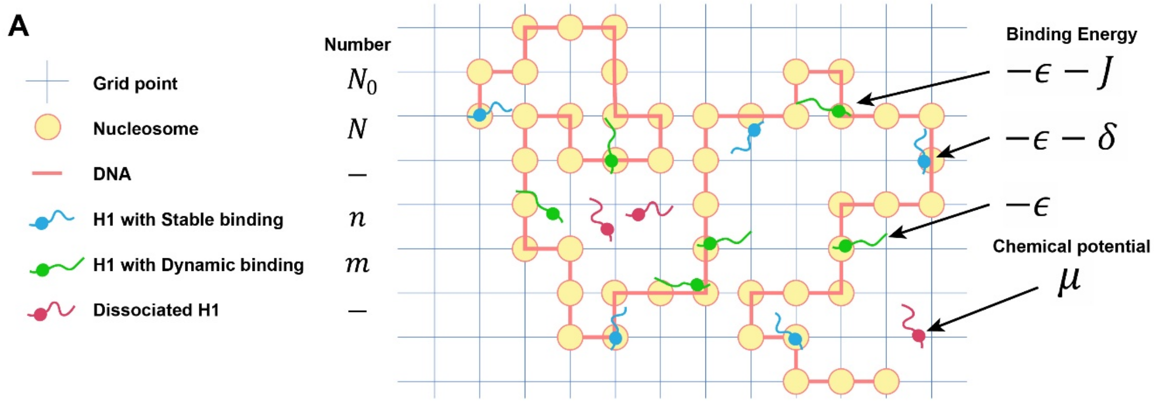

Effective energy:

$$E = -Jm\left[1 - \left(1 - \frac{N}{N_0}\right)^z\right] - (\mu + \epsilon)m - (\mu + \epsilon + \delta)n$$

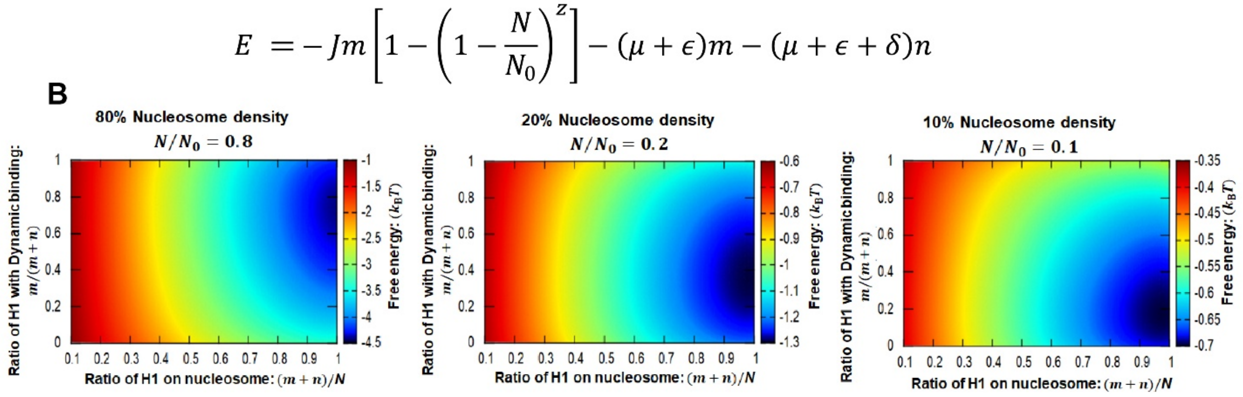

Fig. S9. Multivalent interactions and associating entropy increase stabilize the mobile state of interactions between H1 and nucleosomes.

(A) Schematic representation of the mean-field theory of H1 and nucleosomes (see the Methods for details). N nucleosomes are arranged on N_0 grid points. Of the H1 molecules, n binds to the most stable sites at the nucleosome dyads, with the intrinsically disordered CTD of each H1 interacting with nearby DNA. These H1 molecules are in a low-energy state of $-\epsilon - \delta$ ($\epsilon > 0$, $\delta > 0$). Other m H1 molecules bind near the dyads in higher-energy states of $-\epsilon$. If an H1's intrinsically disordered CTD associates with a nearby nucleosome located within z neighboring grid points, the system stabilizes further by $-J$ per pair. Dissociated H1 molecules, characterized by chemical potential μ, diffuse freely. **(B)** Free energy plot with different nucleosome densities (80%, 20%, 10%). With denser nucleosomes, a higher ratio of H1 with dynamic binding is favored.

Movie S1.

MD simulation of a 12-mer nucleosome array with 12 linker histone H1 molecules. The nucleosome repeat length (NRL) is 165 bp. In the presence of H1, the nucleosome array adopts an irregular folding pattern, with H1 dynamically interacting with multiple nucleosomes.

Movie S2.

MD simulation of a 12-mer nucleosome array (195 bp NRL) with 12 linker histone H1 molecules. In the presence of H1, the nucleosome array adopts an irregular folding pattern, with H1 dynamically interacting with multiple nucleosomes.

Movie S3.

Live-cell imaging of single nucleosomes labeled with TMR in an RPE-1 cell (50 ms per frame). Clear, well-separated dots are observed, each exhibiting single-step photobleaching, indicating that each dot represents a single H2B-Halo-TMR molecule within an individual nucleosome. The scale bar is 5 μm.

Movie S4.

Live-cell imaging of single H1.2 molecules labeled with TMR in an RPE-1 cell (50 ms per frame). Clear, well-separated dots are observed, each exhibiting single-step photobleaching after background subtraction (see Fig. 2E). This indicates that each dot represents a single H1.2-Halo-TMR molecule associated with chromatin. The scale bar is 5 μm.

Movie S5.

Dual-color live-cell imaging of single H2B-PAmCherry (left) and single H1.2 molecules labeled with PA-JF646 (right) in an RPE-1 cell (50 ms per frame). After PALM reconstruction and tracking, images in Movie S6 were obtained.

Movie S6.

Movie of single H1.2 molecules labeled with PA-JF646 in an RPE-1 cell (50 ms per frame). The determined position of each H1.2 molecule was shown as a green particle, while recent trajectories were depicted in cyan. Chromatin domain structures were visualized in magenta using PALM imaging of H2B-PAmCherry. Notably, the “liquid-like” motion of H1 is predominantly observed within chromatin domains.

Movie S7.

MD simulation of 108-mer nucleosome (195 bp NRL) cluster plus 108 Linker Histone H1. As the simulation progresses, the nucleosome cluster adopts a more irregular structure, with H1 dynamically diffusing within the cluster (see Figs. 5A and S6A).
